# Comparative Venom Multiomics Reveal the Molecular Mechanisms Driving Adaptation to Diverse Predator–Prey Ecosystems in Closely Related Sea Snakes

**DOI:** 10.1093/molbev/msad125

**Published:** 2023-06-05

**Authors:** Hao Zheng, Junjie Wang, Hairong Fan, Shuocun Wang, Ruiwei Ye, Linxue Li, Sheng Wang, An Li, Yiming Lu

**Affiliations:** Department of Critical Care Medicine, Shanghai Tenth People's Hospital, School of Medicine, Tongji University, Shanghai, China; School of Medicine, Shanghai University, Shanghai, China; Department of Critical Care Medicine, Shanghai Tenth People's Hospital, School of Medicine, Tongji University, Shanghai, China; Department of Critical Care Medicine, Shanghai Tenth People's Hospital, School of Medicine, Tongji University, Shanghai, China; School of Medicine, Shanghai University, Shanghai, China; School of Medicine, Shanghai University, Shanghai, China; School of Medicine, Shanghai University, Shanghai, China; Department of Critical Care Medicine, Shanghai Tenth People's Hospital, School of Medicine, Tongji University, Shanghai, China; Department of Critical Care Medicine, Shanghai Tenth People's Hospital, School of Medicine, Tongji University, Shanghai, China; Department of Health Toxicology, Faculty of Naval Medicine, Naval Medical University, Shanghai, China; Department of Critical Care Medicine, Shanghai Tenth People's Hospital, School of Medicine, Tongji University, Shanghai, China; School of Medicine, Shanghai University, Shanghai, China; Department of Biochemical Pharmacy, School of Pharmacy, Naval Medical University, Shanghai, China

**Keywords:** multiomics, biodiversity, predator–prey, diet divergence, ncRNAs

## Abstract

Predator–prey arms races are ideal models for studying the natural selection and adaptive evolution that drive the formation of biological diversity. For venomous snakes, venom is a key bridge linking snakes with their prey, but whether and how venom evolves under the selection of diet remains unclear. Here, we focused on two closely related sea snakes, *Hydrophis cyanocinctus* and *Hydrophis curtus*, which show significant differences in prey preferences. Data-independent acquisition (DIA)–based proteomic analysis revealed different degrees of homogeneity in the venom composition of the two snakes, which was consistent with the differential phylogenetic diversity of their prey. By investigating the sequences and structures of three-finger toxins (3FTx), a predominant toxin family in elapid venom, we identified significant differences between the two sea snakes in the binding activity of 3FTx to receptors from different prey populations, which could explain the trophic specialization of *H. cyanocinctus*. Furthermore, we performed integrated multiomic profiling of the transcriptomes, microRNAs (miRNAs), long noncoding RNAs (lncRNAs), and proteomes of the venom glands; constructed venom-related mRNA–miRNA–lncRNA networks; and identified a series of noncoding RNAs involved in the regulation of toxin gene expression in the two species. These findings are highly informative for elucidating the molecular basis and regulatory mechanisms that account for discrepant venom evolution in response to divergent diets in closely related snakes, providing valuable evidence for the study of coselection and coevolution in predator–prey ecosystems.

## Introduction

Biodiversity is one of the most striking features of life on Earth, as species originating from the same ancestor may have evolved in different directions during their evolution. Since the diversity of species and their traits is facilitated by natural selection from interactions with other species or the environment (Patel 2006), predator–prey ecosystems, especially the arms races between venomous snakes and their prey, are good models for studying evolutionary diversity. The processes and mechanisms that drive the diverse evolution of snake venom, particularly the role of diet, have been the subject of intense interest among toxin researchers.

For venomous snakes and their prey, venom is a direct mediator of predator–prey interactions and shows variations among taxa. It is widely acknowledged that the primary function of snake venom is to facilitate prey capture and digestion; however, the extent to which the prey drives the evolution of the venom variety has been controversial. Although some scholars have argued that venom diversity is largely the result of neutral evolutionary processes (Sasa 1999; Mebs 2001), most scientists believe that snake venom components are subjected to strong natural selection from specific feeding niches (Barlow et al. 2009; Ji et al. 2018; Holding et al. 2021; Xie et al. 2022). The recurrent confrontation between venomous snakes and their prey is supposed to generate coevolutionary forces, driven by which prey evolves defensive strategies that make them resistant to snake venoms; snakes are constantly pressured to optimize and adjust the toxins in their venoms. This process of the arms race could produce mutually selective effects that eventually lead to genotypic and phenotypic changes involving prey toxin receptors (Tarvin et al. 2017; Ji et al. 2018) and toxin families and snake venom composition (Daltry et al. 1996; Sasa 1999; Kordis and Gubensek 2000). The complexity of venom composition was recently reported to be positively associated with prey phylogenetic diversity rather than with the overall number of prey species alone in a large clade of North American pit vipers, which strengthened the evidence of venom evolution under selection from the diet (Holding et al. 2021).

Trait diversification in response to varied food resources can also occur in closely related species (Lamichhaney et al. 2015). *Hydrophis cyanocinctus* (annulated sea snake) and *Hydrophis curtus* (spine-bellied sea snake), two fully aquatic sea snakes (Elapidae: Hydrophiinae) diverging ∼9.5 million years ago, share similar general phenotypic traits, while showing great variations in prey selection (Li et al. 2021). Expectedly, the venom-related molecular traits displayed significant divergence. In the aspect of toxin families, we previously found a marked discrepancy (20 vs. 10) in the gene copy number of three-finger toxins (3FTx) in the genomes of *H. cyanocinctus* and *H. curtus*, which also had a dosage effect on the expression of the 3FTx family at mRNA and protein levels (Li et al. 2021). Since 3FTx is the principal toxin family in the venom of Elapidae snakes and acts as the most lethal weapon with neurotoxicity for elapid foraging, its multilevel differences were considered to be associated with different prey preferences between the two sea snakes. However, the specific manner in which 3FTx molecules are functionally fitted to their diverse prey has not been investigated. On the other hand, the composition of 3FTx and other toxin-related proteins/peptides may also contribute to the overall toxicity of snake venom. Several groups have reported the venom gland transcripts and venom proteins of *H. cyanocinctus* and *H. curtus*, but the correlation between their venom composition and feeding strategies has not been comparatively studied (Tan et al. 2019; Wang et al. 2020; Tan and Tan 2021; Zhao et al. 2021). Moreover, there is a lack of in-depth understanding of the regulatory mechanisms underlying the varied expression of venom constituents during adaptation to divergent diets.

The expression of protein-coding genes in specific tissues or organs is regulated at transcriptional and posttranscriptional levels by multiple elements, among which noncoding RNAs (ncRNAs), including microRNAs (miRNAs) and long noncoding RNAs (lncRNAs), are quite nonnegligible. MiRNAs are a class of small (18–25 nucleotides in length), single-stranded ncRNAs that, in most cases, exert sequence-targeted posttranscriptional repression by inducing the silencing or degradation of mRNA molecules through Watson–Crick base pairing, primarily in the 3′-untranslated region (3′-UTR) (Jonas and Izaurralde 2015; Bartel 2018). lncRNAs are defined as transcripts of at least 200 nucleotides that are not translated into proteins, and they are also found to have the ability to regulate the abundance or activity of other RNAs to which they bind following the principle of complementary base pairing (Kopp and Mendell 2018). Although the functions of miRNAs and lncRNAs in humans and other model organisms have been gradually recognized over the last two decades (Beermann et al. 2016), their roles in specialized species and organs, such as the snake venom gland, remain enigmatic. To date, several studies have covered the unidirectional modulating effects of miRNAs on toxin synthesis in venomous snakes and on muscle growth in nonvenomous snakes (Durban et al. 2013; Durban et al. 2017; Durban et al. 2018; Khan et al. 2020), but only one study has involved the identification of venom-related lncRNAs, which were supposed to encode two functional myotoxins in the prairie rattlesnake (Gopalan et al. 2022). More importantly, as lncRNAs can provide decoy targets for miRNAs and act as competing endogenous RNAs (ceRNAs) (Cesana et al. 2011), the probable interactions among these ceRNAs, miRNAs, and mRNAs in the regulation of venom protein expression are currently poorly understood.

Recent advances in high-throughput sequencing have revved up the development of evolutionary biology and epigenetics on a multiomic scale, settling scores for vital issues concerning speciation and adaptative evolution (Chen et al. 2019; Gemmell et al. 2020; Ocaña-Pallarès et al. 2022). Our group previously obtained two chromosome-level reference genomes for *H. cyanocinctus* and *H. curtus* and systematically annotated their toxin-related genes (Li et al. 2021). In this study, we utilized state-of-the-art proteomic and transcriptomic technologies to link venom-associated genotypes and phenotypes with the diverged dietary traits of the two sea snakes. Data-independent acquisition (DIA) (Zhang et al. 2020) was used to comprehensively and accurately reconstruct the proteomes of venoms and venom glands. We analyzed the sequences and structures of 3FTx proteins expressed in the venom gland and compared their binding potential to the acetylcholine receptors (AChRs) of different prey. The contrasting venom composition and toxin–receptor interaction characteristics would lay a molecular basis for explicating the differentiated prey preferences of the two species. Furthermore, we determined the miRNA and lncRNA profiles of the venom glands and identified candidate toxin-related RNAs implicated in the lncRNA–miRNA–mRNA regulatory networks responsible for differentially expressed toxins in *H. cyanocinctus* and *H. curtus*. Comparative, integrated transcriptome–ncRNA–proteome analysis and functional evaluation from one toxin family to the venom cocktail are expected to elucidate the mechanisms that account for dietary divergence in terms of venom evolution in distinct predator–prey ecosystems.

## Results

### Divergence of the Diet

**Fig. 1.**
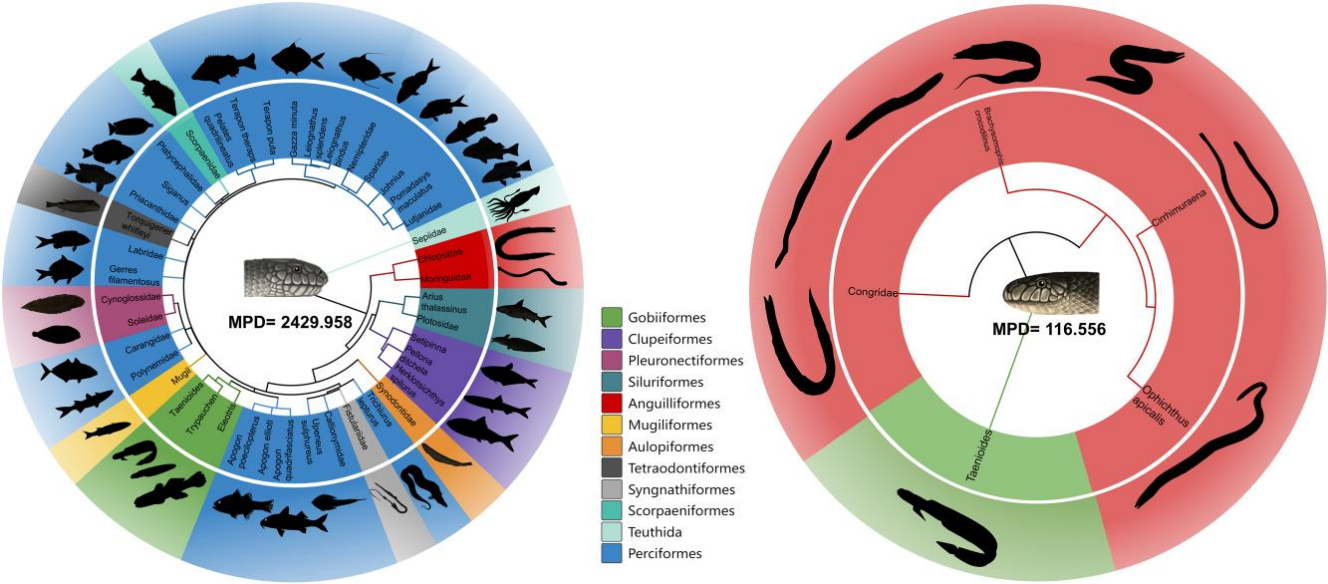
Phylogeny of the prey of *H. curtus* (left) and *H. cyanocinctus* (right). Prey species were collected from previous reports on organisms found in the stomachs of sea snakes. Some prey could only be identified to their superordinate taxa, which are shown as corresponding families or genera. Representative species are displayed in black silhouettes. The phylogeny was recovered using the TimeTree5 (Kumar et al. 2022) online tool (http://www.timetree.org/).

As closely related *Hydrophis* snakes, *H. cyanocinctus* and *H. curtus* are physically similar in several respects, but their feeding habits differ significantly. We summarized the prey species of the two sea snakes based on previous studies (Glodek and Voris 1982; Voris and Voris 1983; Fry et al. 2001; Lobo et al. 2005; Sanders, Rasmussen, et al. 2013; Sherratt et al. 2018) and performed a phylogenetic analysis. As shown in the evolutionary tree (fig. 1), the prey of *H. curtus* covers at least 42 branches from 35 families in 12 orders, including not only vertebrates but also invertebrates such as squids under Decapodiformes. In contrast, the prey of *H. cyanocinctus* comprises only five branches from three families in Anguilliformes (eel) and Gobiiformes (goby). The diet of *H. cyanocinctus* is dominated by eels and eel-like slender fish, whereas *H. curtus* feeds on a variety of benthic, demersal, and pelagic fish from different depths. In addition, the phylogeny of the prey of *H. cyanocinctus* appeared to be relatively simple, with a mean phylogenetic diversity (MPD) of 116.556, whereas that of *H. curtus*, a representative generalist, was far more complex (MPD = 2,429.958).

The differentiation of the trophic ecology of *H. cyanocinctus* and *H. curtus* can be attributed to biogeographical factors. There are many overlapping sea areas in the geographical distribution of the two dominant sea snake species throughout the Indo-Pacific Ocean (Ukuwela et al. 2016) (supplementary table S1 and fig. S1, Supplementary Material online). The shared ecological niches could have resulted in fierce competition for limited food resources in the same habitat during their ancestral times. As a consequence, *H. cyanocinctus* and *H. curtus* have adopted different routes of predation, giving rise to different selection pressure. Over the long-term evolution under natural selection, the venom of *H. curtus* is likely to have adapted to the generalist hunting style, whereas the venom of *H. cyanocinctus* might have become more specialized, with stronger toxicity toward specific prey to achieve higher hunting efficiency.

### DIA Proteomics Characterization of Venoms and Venom Glands

**Fig. 2.**
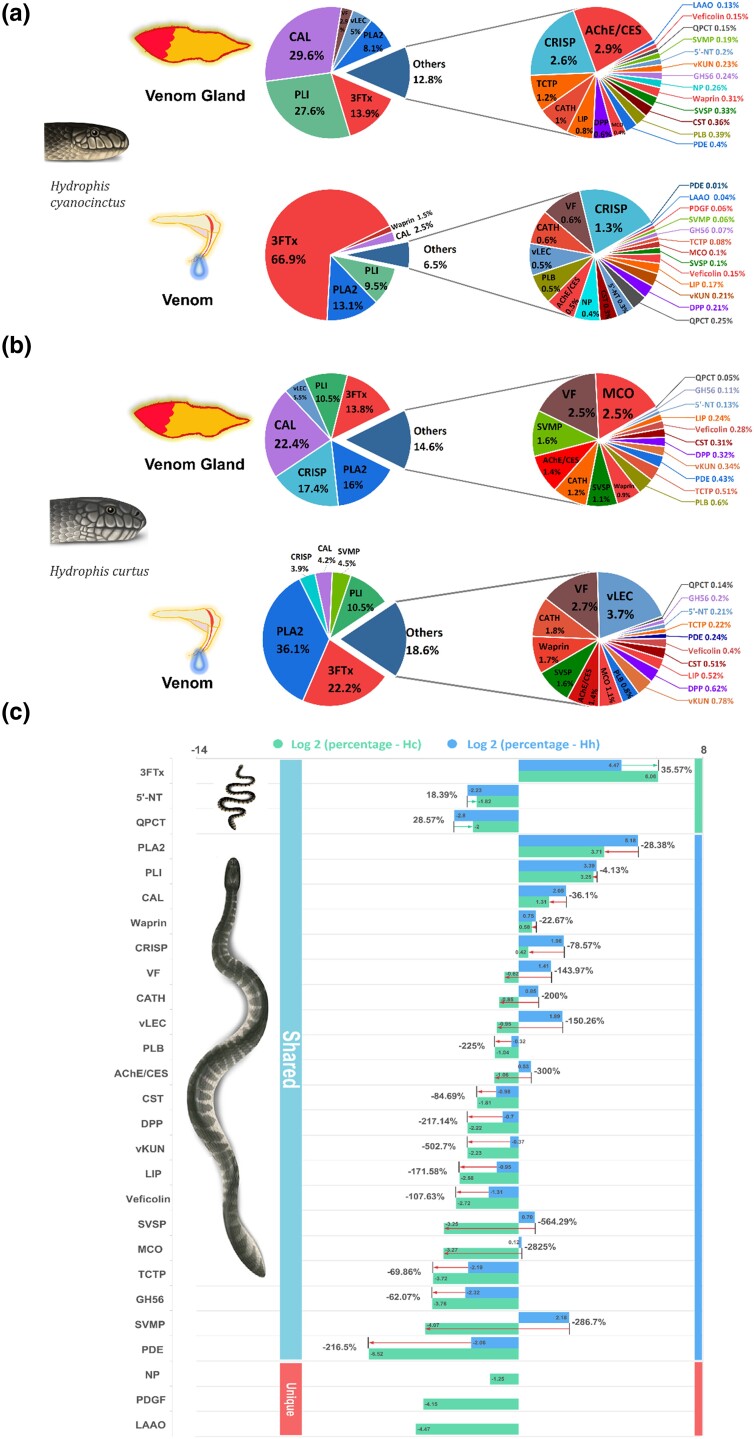
Proteomic characterization of venoms and venom glands of *H. cyanocinctus* and *H. curtus* based on DIA technology. (*a*) Percentage composition of toxin-related protein in the venoms and venom glands of *H. cyanocinctus*. (*b*) Percentage composition of toxin-related protein families in the venoms and venom glands of *H. curtus*. The full names of these families are shown in supplementary table S2, Supplementary Material online. (*c*) Comparison between the relative abundance of toxin-related families in the venoms of the two sea snakes. The abundance percentages were log2 transformed. The arrows between the horizontal bars represent the difference of abundance between the two snakes, and the value is the relative ratio of the difference. The vertical bars on the right represent the range of families that has higher relative abundance in each snake. Hc, *H. cyanocinctus*; Hh, *H. curtus*.

In order to explore venom evolution under the selection of divergent diets in sea snakes, we characterized the proteomes of the venoms and venom glands of *H. cyanocinctus* and *H. curtus* using the emerging technique of DIA, which possesses an obvious advantage in sensitively detecting low-abundance proteins in complex samples with high accuracy and reproducibility (Zhang et al. 2020) (supplementary Data Sets S1–S4, Supplementary Material online). In total, 27 and 24 toxin-related protein families were identified in the venomics of *H. cyanocinctus* and *H. curtus*, respectively. There were 24 families shared between the two species, represented by 3FTx, phospholipase A2 (PLA2), phospholipase A2 inhibitor (PLI), cysteine-rich secretory protein (CRISP), calmodulin (CAL), snake venom metalloproteinase (SVMP), true venom lectin (vLEC), and venom factor (VF) (fig. 2*a* and *b* and supplementary table S2, Supplementary Material online). The families of neuropeptide (NP), L-amino acid oxidase (LAAO), and platelet-derived growth factor (PDGF) exhibited low abundance (0.04–0.4%) but were specific to the venomics of *H. cyanocinctus* (supplementary fig. S2, Supplementary Material online). Since they have similar gene copy numbers in the genomes of the two snakes (Li et al. 2021), this endemism might not represent the essential difference in venom composition between *H. curtus* and *H. cyanocinctus*; however, their biological functions in sea snake venom need to be further investigated. For *H. curtus*, the venom gland harbored the same kinds of toxin-associated protein families as the venom. For *H. cyanocinctus*, the PDGF family was unique to its venom, which might be due to the inapplicability of detection of extremely low abundance (<0.01%) in venom gland and the concentrating effect in venom milking. Interestingly, the PLI family was found in both the venom gland (10.5–27.6%) and venom (9.5–10.5%) proteomes, and so did several other toxin-related (but not toxic) proteins such as CAL. The roles these venom proteins play in the predation of sea snakes are worth studying in depth.

Through quantitative analysis, we identified variances in the abundance ratio of these toxin-related proteins between the two snakes. In *H. cyanocinctus* venom, 3FTx family occupied the highest portion of 67%, and the proportion difference between 3FTx and any other family was >50%, with 19 families each accounting for <1%. In contrast, 3FTx (22.3%) and PLA2 (36.3%) constituted the majority of *H. curtus* venom, and the percentage of 13 families each was >1% (fig. 2*c*). By interspecific comparison of each toxin-related family, we found that the relative abundances of only three families, 3FTx and two lowly expressed families, glutaminyl-peptide cyclotransferase (QPCT) and 5′-nucleotidase (5′-NT), were higher in *H. cyanocinctus* than those in *H. curtus* (fig. 2*c*). Therefore, the venom composition of *H. cyanocinctus* was dominated by 3FTx and was more concentrated, whereas the composition of *H. curtus* venom was relatively more balanced. These proteomic features indicate that *H. cyanocinctus* relies mainly on 3FTx for foraging, whereas *H. curtus* requires a combination of various toxin-related proteins, which might have a bearing on its wide prey range, including more species and higher diversity (fig. 1).

Since the venom composition of *H. cyanocinctus* and *H. curtus* also displays a 3FTx/PLA2 dichotomic feature similar to that of some Hydrophiinae and Elapinae snakes, we compared the reported venom proteome and diet of five sea snakes (supplementary fig. S3, Supplementary Material online). Interestingly, snakes with 3FTx-dominant venom tended to have a relatively more concentrated diet unique to two to four prey families, especially eel- and goby-like fishes, while species with PLA2-dominant venom show broader and more diverse prey ranges. As the selected sea snakes have shared ecological niches in biogeography, this phenomenon might support that venom variations in 3FTx/PLA2 composition are correlated with the selection pressure from various dietary habits in sea snakes, although further investigations including more Hydrophiinae species and more quantitative analyses are needed.

### Variations of 3FTx in Sequence, Structure, and Function

Most snake venom toxins target specific receptors and provoke harmful effects on the physiological systems of animals, allowing snakes to hunt and kill their prey efficiently or escape from natural enemies. Therefore, the receptor-binding activity of a toxin directly affects its toxicity toward the prey. 3FTx belongs to a family of α-neurotoxins that cause paralysis by binding to the nicotinic acetylcholine receptors (nAChRs) at the postsynaptic region of the neuromuscular junction (Phui Yee et al. 2004). Since 3FTx was observed to be the mainstay in the venom proteome of *H. cyanocinctus* and was reported to be more toxic than PLA2 in elapid venom, we investigated whether and how 3FTx could be associated with its simple diet in terms of molecular structure and function.

**Fig. 3.**
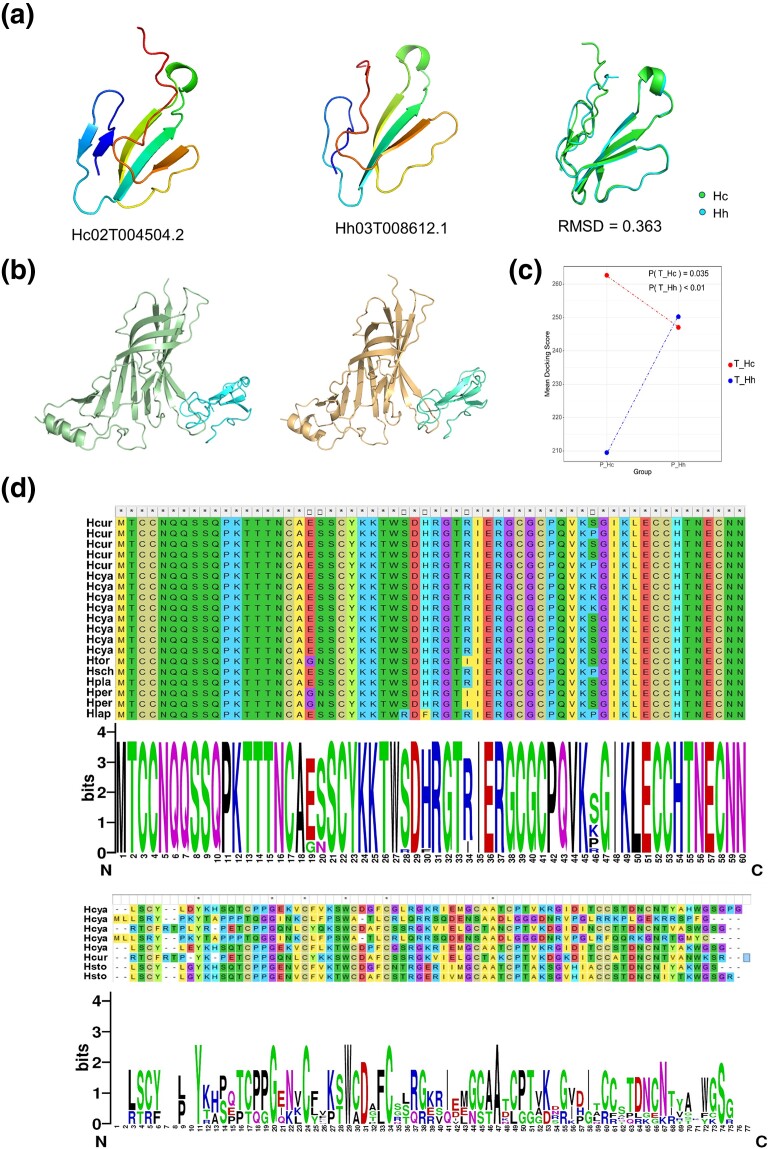
Divergent evolution of the sequence, structure and function of 3FTx between *H. cyanocinctus* and *H. curtus*. (*a*) Comparison of the structure models of the most highly expressed 3FTx proteins (Hc02T004504.2 and Hh03T008612.1) in the venom of the two snakes. RMSD, root mean square deviation. (*b*) Simulated structures of two nAchR α-subunits (extracellular domains) bound to two highly expressed 3FTx proteins (Hh03T008612.1 and Hc03T010178.5) in the venom of the two snakes. The receptor in the left schematic is from *S. maximus*, a prey of *H. curtus*; the receptor in the right schematic is from *Periophthalmus gracilis*, a prey of *H. cyanocinctus*. (*c*) Dot plot of the docking scores of 3FTx proteins from the two sea snake species with receptors from different prey sources. T_Hc, 3FTx of *H. cyanocinctus*; P_Hc, receptors of *H. cyanocinctus* prey; T_Hh, 3FTx of *H. curtus*; P_Hh, receptors of *H. curtus* prey. The significance of difference was determined using *t*-test. Protein structures were predicted by AlphaFold2-Colab. Molecular docking was performed with the HDOCK server. (*d*) Sequence alignment and LOGO plots of 3FTx-SNTX (upper panel) and 3FTx-LNTX (lower panel) from eight sea snakes. Hcya, *H. cyanocinctus*; Hcur, *H. curtus*; Htor, *Hydrophis torquatus*; Hsch, *Hydrophis schistosus*; Hpla, *Hydrophis platurus*; Hper, *Hydrophis peronii*; Hlap, *Hydrophis lapemoides*; Hsto, *Hydrophis stokesii*.

Via AlphaFold2 (Jumper et al. 2021), we constructed the three-dimensional structure models of the 3FTx proteins expressed in the venom of the two sea snakes, with their sequences derived from our previous genome annotations. The structures of the two most highly expressed 3FTx proteins in *H. cyanocinctus* and *H. curtus* are globally similar with a root mean square deviation (RMSD) of 0.363 Å (fig. 3*a* and supplementary fig. S4, Supplementary Material online). Meanwhile, we performed structure prediction of the nAchRs alpha-subunits (extracellular) of representative prey species of the two sea snakes (supplementary fig. S5, Supplementary Material online). The structures of 3FTx were then separately docked to those of the receptors (fig. 3*b*). Comparison of the docking scores showed that *H. cyanocinctus* 3FTx had significantly (*P* < 0.05) higher binding affinity to the receptors of its own prey than to those of *H. curtus* prey (fig. 3*c* and supplementary tables S3 and S4, Supplementary Material online). This suggested that the toxic function of 3FTx proteins in *H. cyanocinctus* venom may have evolved directionally to adapt to its specific diet, with a narrower prey range but more efficient utilization of toxins.

As the binding of a toxin protein to its target is established depending on the contact of several amino acid residues, mutations in these key sites can influence the local structure or interaction mode and eventually affect the receptor-binding ability of the toxin. We performed multiple sequence alignment of the 3FTx proteins of several sea snakes of known diet and found that the sequences of short-chain neurotoxins (SNTX) are relatively conserved, except for the 46th site, which was supposed to be one of the receptor-binding sites of SNTX (Nys et al. 2022). The residues S (serine) and P (proline) at this locus appear in SNTX sequences of all the selected snakes, while the variations of K (lysine) and R (arginine) at this site seem to be unique in *H. cyanocinctus* (fig. 3*d*). In contrast, the sequences of long-chain neurotoxins (LNTX) appeared markedly variable, in both *H. cyanocinctus* and among the selected species. Many mutations arose at the residues including 6th, 9th, 29th, 66th, and 68^th^, which were identified as important binding sites of LNTX (Rahman et al. 2020). It is noteworthy that the LNTX subfamily made up the majority of 3FTx expressed in the venoms of both snakes (supplementary Data Sets S1 and S2, Supplementary Material online). Therefore, it is possible that the divergent evolution of LNTX contributed more to the differences in the receptor-binding activity of 3FTx between the two sea snakes, thereby leading to distinct toxic effects of their venoms on different prey populations. Additionally, we evaluated the evolutionary rates of 3FTx family in *H. cyanocinctus* by calculating the number of synonymous (Ks) and nonsynonymous (Ka) nucleotide substitutions between toxin gene pairs. The Ka/Ks ratio of LNTX subfamily presented significantly higher than that of SNTX subfamily (supplementary fig. S6, Supplementary Material online), indicating that the LNTX genes of the annulated sea snake might have undergone stronger selection and rapid evolution to acquire continuous functional improvement and achieve optimal toxicity toward a specialized diet.

### Multiomic Profiling of Venom Glands

The above results provide a certain degree of molecular evidence that links diverged venom evolution with differences in predation between the two sea snakes. Further, we attempted to explore the intrinsic regulatory mechanisms responsible for the distinction in venom composition through multiomics. By high-throughput sequencing and bioinformatic analyses, the transcriptomes, miRNAs, and lncRNAs present in the venom glands of the two snakes were deciphered (supplementary tables S5 and S6 and figs. S7–S10, Supplementary Material online). In *H. cyanocinctus*, 44,135 mRNAs, 964 miRNAs, and 2,855 lncRNAs were identified and quantified, of which 532 miRNAs and 410 lncRNAs were newly discovered. In *H. curtus*, 36,583 mRNAs, 855 miRNAs, and 3,047 lncRNAs were characterized, of which 422 miRNAs and 835 lncRNAs were newly predicted. There were 432 miRNAs in *H. cyanocinctus* and 433 miRNAs in *H. curtus* that matched to known sequences of *Ophiophagus hannah* (king cobra) and *Python bivittatus* (Burmese python) in miRBase (supplementary Data Sets S5–S10, Supplementary Material online).

In terms of transcript quantification, we noticed differences in the distribution of mRNA abundances in the venom glands between *H. cyanocinctus* and *H. curtus*. The number of lowly expressed mRNA transcripts with fragments per kilobase of transcript per million mapped reads (FPKM) < 0.5 in *H. cyanocinctus* was 8,020 (38%) more than that in *H. curtus*; the number of highly expressed mRNA transcripts with FPKM ≥ 10 in *H. cyanocinctus* was 1,289 (47%) fewer than that in *H. curtus*. However, there were no significant differences in the expression distribution of miRNAs and lncRNAs between the two sea snakes (supplementary fig. S11, Supplementary Material online).

### Regulatory Networks of Gene Expression in the Venom Glands

**Fig. 4.**
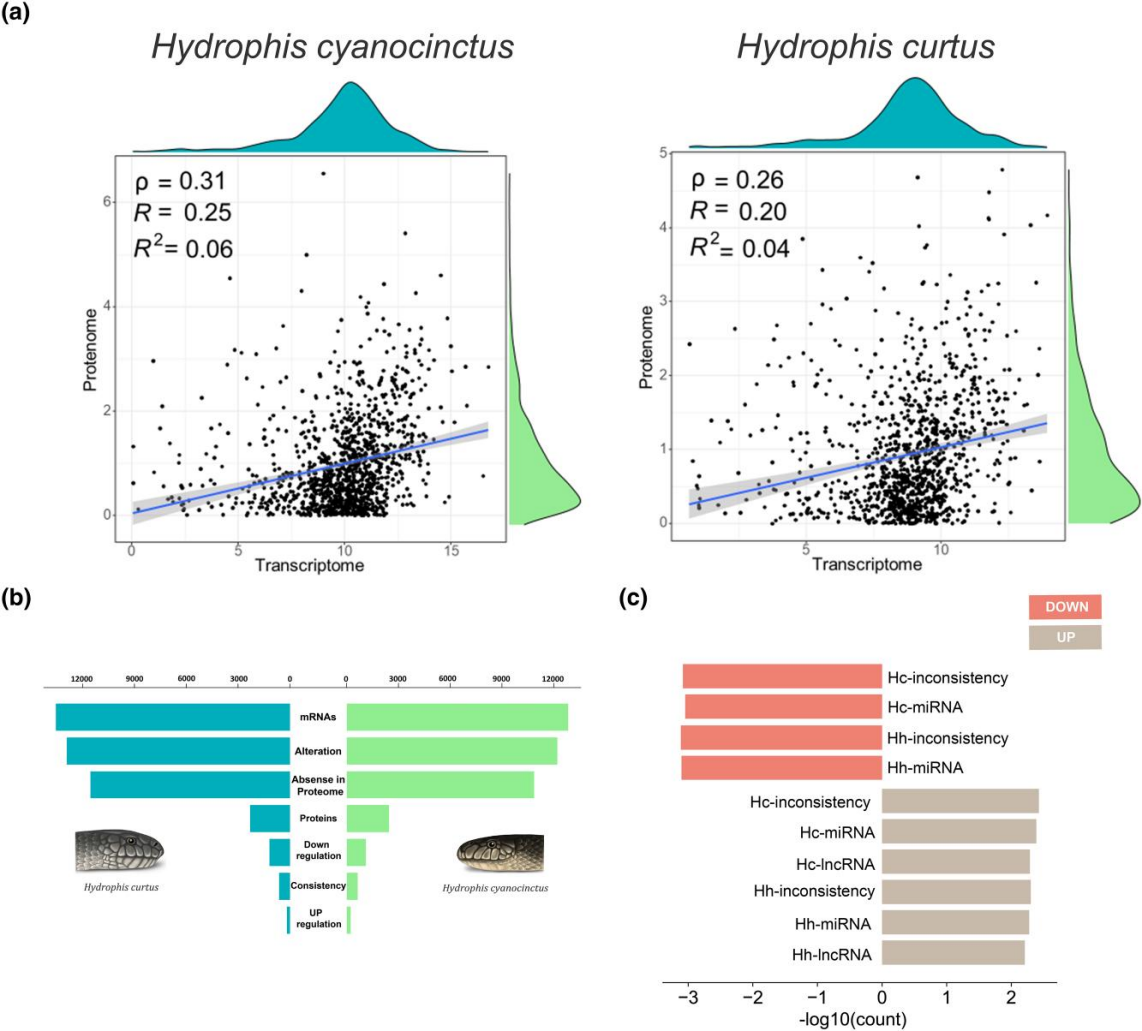
Combined analysis of transcriptome and proteome in the venom glands of *H. cyanocinctus* and *H. curtus*. (*a*) Correlation analysis of transcriptome and proteome expression levels in *H. cyanocinctus* and *H. curtus*. (*b*) Quantitative statistics of the transcripts, proteins, and their expression level changes in the two snake venom glands. (*c*) Statistics on the number of downregulated mRNAs in the two snake venom glands. Hc inconsistency represents mRNAs with changed expression levels from transcriptome to proteome in the venom gland of *H. cyanocinctus*. Hc-miRNA represents mRNAs with changed expression levels that were regulated by miRNAs. Hc-lncRNA represents mRNAs with changed expression levels that were regulated by lncRNAs.

In the genetic information flow from transcriptome to proteome, the expression levels of mRNAs and proteins are not highly uniform in most cases, resulting from multiple levels of regulation (Vogel and Marcotte 2012). The transcriptome–proteome correlation analysis revealed that the consistency between the expression levels of mRNAs and proteins from transcription to translation was poor in the venom glands of both snakes, with the Pearson's correlation coefficients (*R*) of 0.20–0.25 (fig. 4*a* and supplementary fig. S12, Supplementary Material online). We counted and compared the number of expressed mRNA transcripts, proteins, and upregulation and downregulation levels in the venom glands of the two sea snakes (fig. 4*b*). For *H. cyanocinctus*, a total of 14,025 mRNAs (FPKM > 1) were screened in the transcriptome, and 2,669 proteins were obtained in the proteome. Through comparative analysis of the two data sets, we identified 686 mRNAs with basically consistent expression during translation and 13,339 mRNAs with altered expression levels. The corresponding changes reflected in the proteomes were divided into three categories including 11,865 proteins that were not expressed or detected, 1,207 proteins with decreased expression, and 267 proteins with increased expression. The data for *H. curtus* were similar, with 14,837 mRNAs (FPKM > 1) screened in the transcriptome and 2,528 proteins obtained in the proteome. Among these, there were 691 mRNAs with no significant alteration in expression during translation, 14,146 mRNAs with changed expression levels, 12,645 proteins not expressed or detected, 1,301 proteins with decreased expression, and 200 proteins with increased expression.

Through functional enrichment analysis, we found that genes with altered expression were involved in important biological functions and pathways essential for cell growth (supplementary fig. S13 and Data Sets S11–S14, Supplementary Material online). For example, protein synthesis (GO: 0044877), processing and secretion (GO: 0016192), and the corresponding energy metabolism were closely related to toxin synthesis and secretion in snake venom glands. Protein synthesis requires nitrogen sources as raw materials, so the cells need to metabolize organic nitrogenous compounds (GO: 0006520). In addition, the synthesis and secretion of proteins involved a series of biological processes, such as protein-binding interactions (GO: 0005509, GO: 0005525, GO: 0008135, and GO: 0090079), activation of protein activity (GO: 0003824, GO: 0016853, and GO: 0016491), and vesicle-mediated transport of the synthesized proteins (GO: 1903561 and GO: 0070062). To seek for the possible regulatory action of ncRNAs on mRNA–protein expression levels from transcriptome to proteome in the venom glands, we conducted target gene prediction of miRNAs and identified miRNAs and lncRNAs that could directly or indirectly affect mRNA translation and regulate protein expression in the venom glands (fig. 4*c* and supplementary Data Sets S15–S18, Supplementary Material online). We then performed ceRNA analysis and the top 100 mRNA–miRNA–lncRNA pairs with the highest MuTATE scores were selected to map the regulatory networks (supplementary fig. S14 and Data Sets S19 and S20, Supplementary Material online).

### Regulation of Toxin Expression by miRNAs and lncRNAs

**Fig. 5.**
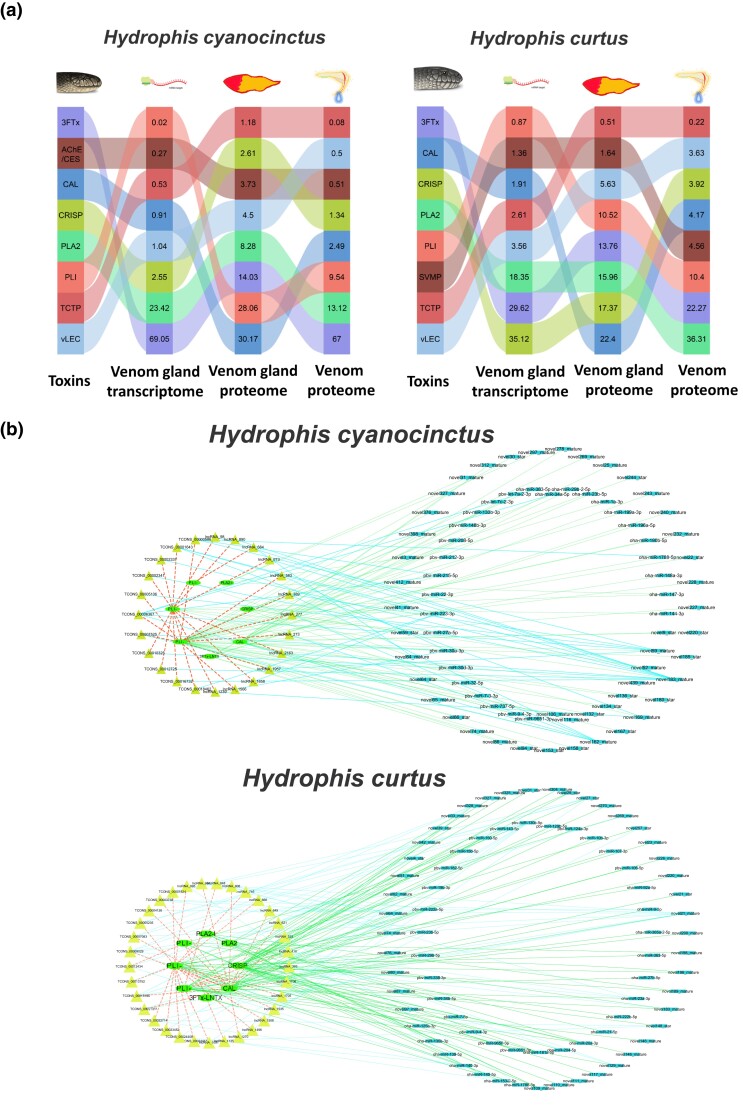
Expression level changes and regulation of toxins in *H. cyanocinctus* and *H. curtus*. (*a*) Sankey diagram of abundance shift of major toxin families in the venom gland transcriptome, venom gland proteome, and venom proteome. (*b*) Regulatory networks of toxin-related mRNAs, miRNAs, and lncRNAs in the venom glands.

The expression of toxins and related genes in venom glands is regulated in various ways. We analyzed the abundance shifts of major toxin-related families in the two sea snakes at each stage of transcription, synthesis, and secretion, which correspond to the venom gland transcriptome, venom gland proteome, and venom proteome, respectively (fig. 5*a*). The relative abundances of toxin activity-regulating proteins, such as PLI, CAL, and AchE/CES, were greatly reduced from the venom gland to the venom, whereas 3FTx, PLA2, and other toxins were concentrated in the process of venom secretion. For individual toxins from transcriptome to proteome in the venom gland of *H. cyanocinctus*, PLI-α, PLI-β, and AChE/CES genes were upregulated with the rankings increasing by a total of 199.13%; PLI-γ and PLA2-I genes were downregulated with their rankings decreasing by a total of 55.46%. In the venom gland of *H. curtus*, six toxin-related genes were upregulated (AChE/CES, PLI-α, PLI-β, PLI-γ, vLEC, and CAL) with the rankings increasing by a total of 233.6%, and five genes (CRISP, TCTP, 3FTx-LNTX, and PLA2-I) were downregulated with their rankings decreasing by a total of 39.19% from transcriptome to proteome (supplementary fig. S15, Supplementary Material online).

**Fig. 6.**
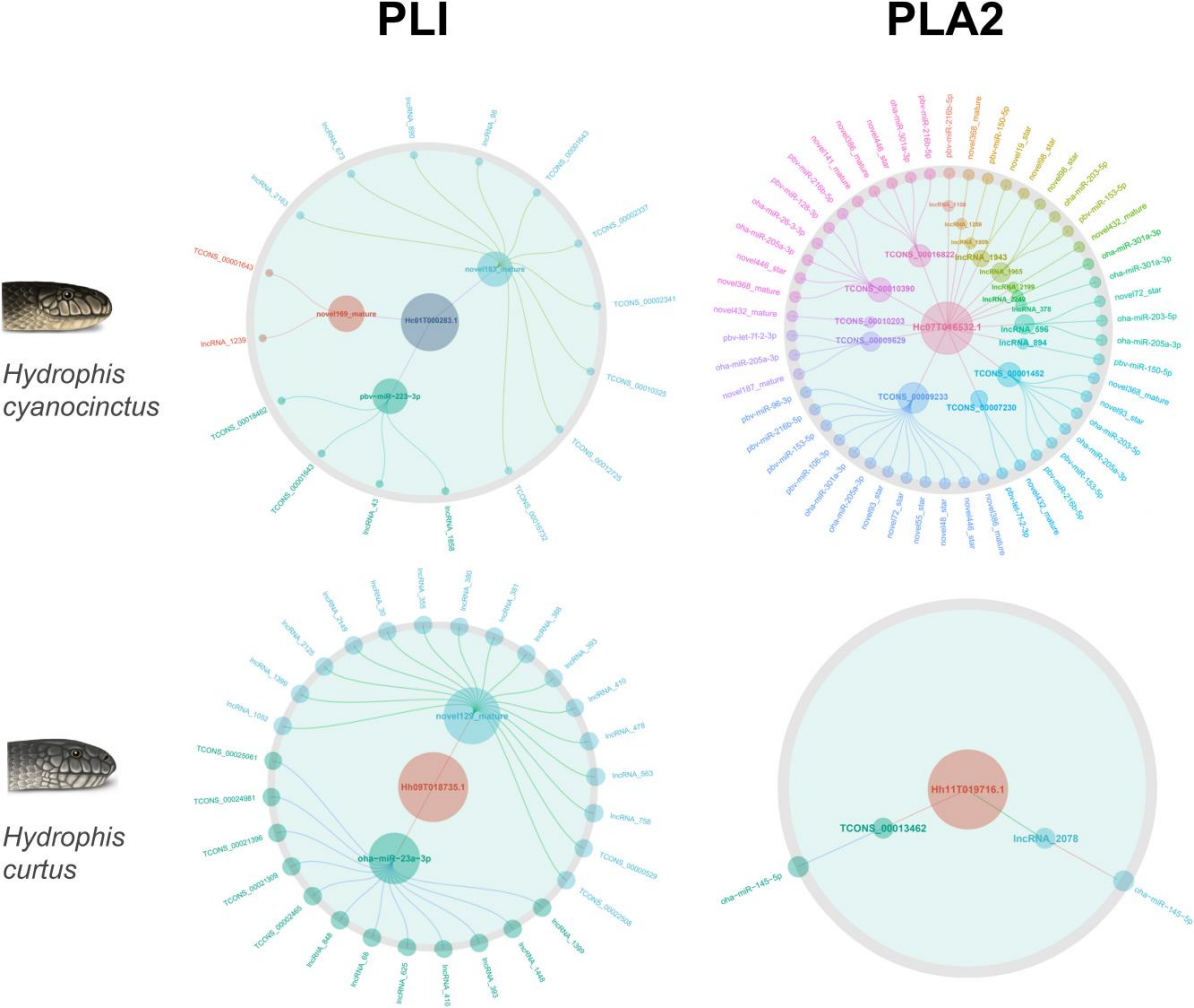
lncRNA–miRNA–mRNA regulatory networks of representative PLA2 and PLI genes of the two sea snakes. The number inside the central circle in each network represents the gene ID. For the networks of PLA2, miRNAs are displayed in the outermost layer, and lncRNAs are displayed in the inner layer. In contrast, the networks of PLI show lncRNAs in the outer layer and miRNAs in the inner layer.

Through advanced mining and analysis of the ceRNA data, we found that most toxin-related families in both sea snakes were regulated by miRNAs and lncRNAs. These toxin-related genes were laid within complex networks involving significantly more ncRNAs than mRNAs, since one toxin-related gene could be linked to several miRNAs or lncRNAs (fig. 5*b*). To further explore the regulation of individual toxins by ncRNAs, we focused on PLA2, a major toxin family with differential expression (13.12% vs. 36.31%) in the two snake venoms, and PLI, the key inhibitors of PLA2 that exhibited significant upregulation from transcriptome (0.02–0.87%) to proteome (10.52–28.06%) in both sea snakes (fig. 5*a*). Interestingly, we noticed that PLA2 and PLI showed mRNA–miRNA–lncRNA regulation patterns that were markedly distinct from each other but conformed to their change of expression levels (fig. 6). The PLA2 mRNA Hc07T016532.1 in *H. cyanocinctus* was regulated by 23 miRNAs but fewer (17) lncRNAs, while Hh11T019716.1 in *H. curtus* was influenced by only one miRNA and two lncRNAs. In contrast, the selected PLI mRNAs in both snakes were regulated by only two to three miRNAs but apparently more (13–25) lncRNAs. Given that lncRNAs could compete with mRNAs for binding to miRNAs and weaken the silencing effect of miRNAs, the difference in number and relative scale of miRNA/lncRNA might partly explain the transcriptome–proteome expression shift (downregulation or upregulation) of major toxins and their different proportions in venom between species. Accordingly, these results indicated that the ceRNA regulatory mechanisms seemed globally conserved but were locally varied among toxin families between *H. cyanocinctus* and *H. curtus*, which may to a certain extent account for their divergent venom composition.

## Discussion

True sea snakes (Hydrophiinae: Hydrophiini) are a young clade of over 60 species that have undergone rapid adaptive radiation and exhibit morphological and ecological diversification (Sanders et al. 2013). The sea snakes *H. cyanocinctus* and *H. curtus* are close relatives of the same genus but show great differences in feeding strategies. By comparative analysis of DIA–based venom proteomics, we found that most of the toxin families expressed in the venom were common to both sea snakes, but the venom compositions of these toxins differed significantly between the two species. The higher degree of uniformity of toxin proportions in *H. curtus* venom is likely associated with its far wider prey range with many more species and higher phylogenetic diversity. We speculate that as different toxins have different toxic effects, venomous snakes tend to make venom components more balanced or complex to diversify the functions of the toxin mixture and enhance their adaptability to diverse prey. This is partly in accordance with and may provide complementary evidence to previous research that correlated the complexity of venom composition with the phylogenetic diversity of prey in pit vipers (Holding et al. 2021).

Since the venom composition of *H. cyanocinctus* is relatively simple and dominated by the 3FTx family, we explored whether this is associated with its trophic specialization. Structure simulation and molecular interaction analysis of toxin–receptor showed that 3FTx of *H. cyanocinctus* possessed significantly higher binding activity to the nAChRs of its own prey, which might represent higher prey-specific toxic activity. Multiple sequence alignment of the 3FTx-SNTX and 3FTx-LNTX subfamilies revealed a series of mutations at the key receptor-binding sites of LNTX, which were considered to lead to variations in the affinity of the toxins with receptors from different prey populations between the two sea snakes. The high variability of gene structure and function together with the higher Ka/Ks substitution ratio may imply an accelerated rate of evolution and stronger selection for LNTX in *H. cyanocinctus*. As LNTX accounts for the major abundance of 3FTx in its venom, we suggest that *H. cyanocinctus* mainly depends on LNTX evolution to obtain optimal toxicity toward its target receptors and hence adapts to a specialized diet, which include eels that might be more flexible in moving and harder to immobilize than other fish.

The diversified evolution of biological traits requires not only genetic variations in response to different extrinsic ecological niches but also epigenetic regulatory factors. Therefore, we investigated the functions of ncRNAs in the intrinsic regulatory mechanisms underlying the altered composition of venom toxins between *H. cyanocinctus* and *H. curtus*. We performed integrated analysis of transcriptome–ncRNA–proteome in the venom glands of the two snakes and identified numerous miRNAs and lncRNAs, which comprise complex ceRNA networks that regulate the expression of toxins and toxin-related proteins. Given that miRNAs are known negative regulators of gene expression and that lncRNAs have been previously shown to take part in a multitude of biological processes including growth and development, immunity, and tumorigenesis (Chen et al. 2016), our study unveiled novel perspectives of the important roles lncRNAs play in venom regulation, suggesting that lncRNAs could bind to miRNAs to reduce the silencing effect and result in the relative upregulation of toxin genes.

Our previous studies reported the variation in *3FTx* gene copy numbers between the two snakes, with 20 copies of *3FTx* gene in *H. cyanocinctus* and only 10 copies in *H. curtus*, demonstrating part of the reason for the higher percentage of 3FTx in *H. cyanocinctus* venom from a genetic perspective. The different regulatory patterns of toxin expression by miRNAs and lncRNAs at the posttranscriptional level, together with the dosage effect due to copy number variation of toxin genes, to a great extent could explain the alteration in venom composition in the two snakes. Moreover, the divergent venom composition and receptor-binding activity of 3FTx largely determine the discrepant toxicity of venom toward different prey, which has been indirectly reflected in in vivo tests on mice, suggesting that the venom of *H. cyanocinctus* is overall more toxic than that of *H. curtus* (Wang et al. 2020).

Apart from being a biochemical weapon for predation, snake venom is also a natural repository of bioactive molecules for drug discovery (Wang et al. 2016; Zheng et al. 2016); therefore, it is essential to strengthen the conservation of diversity of these sea snakes with high medicinal value. In conclusion, the present study revealed the biological relevance of discrepant trophic ecology and divergent venom evolution in closely related sea snakes in a wide scope of molecular functions and expression regulations based on comprehensive multiomics. We believe that these findings contribute valuable evidence for the study of coselection and coevolution in predator–prey ecosystems and provide new insights on the formation of biodiversity on Earth, although further in-depth investigation is still needed through in vitro and in vivo functional experiments regarding the structure–activity relationship of 3FTx and other toxins with their targets.

## Materials and Methods

### Animals and Samples

A total of three annulated sea snakes (*H. cyanocinctus*, Hc_1, Hc_2, and Hc_3) and three spine-bellied sea snakes (*H. curtus*, Hh_1, Hh_2, and Hh_3) were used in this study, which were captured in the South China Sea adjoining Sanya, China. Venom was extracted from the snakes 3 days prior to sacrifice. Venom gland tissues were subsequently taken, and the samples were stored at −80 °C until use.

Total RNA was extracted from the ground venom gland, and the quality was checked for purity, concentration, and integrity (RNA integrity number [RIN]). The samples with RIN ≥ 7 were subjected to subsequent library construction. Next, we used a ribo-zero kit to deplete ribosomal RNA, added the interruption reagent to interrupt the RNA into short fragments, used the interrupted RNA as the template, synthesized the single-stranded cDNA with six-base random primers, and then prepared a two-stranded synthesis reaction system to synthesize the two-stranded cDNA. The cDNA strand was then digested using the UNG enzyme method, and only the cDNA strand with different connectors was retained. The cDNA strand was purified using the kit; the purified cDNA strand was then end-repaired, A-tailed, and connected to the sequencing connector, followed by fragment size selection, and finally PCR amplification. After quality check by Agilent 2100 Bioanalyzer, the library was sequenced on Illumina HiSeq X Ten sequencer with a sequencing depth of ∼100×.

### Transcript Quantification

RNA extracted from the venomous gland tissue was sequenced using an Illumina sequencer after rRNA removal, fragmentation, reverse transcription, end-complementation, and PCR amplification. In the transcriptome sequencing analysis, we used reference transcripts as libraries and bowtie2 (Zhang et al. 2021) and eXpress software (Roberts et al. 2011; Roberts and Pachter 2013) to estimate gene expression levels based on the number of sequenced sequences (reads) localized to the exon regions of the transcripts. The mRNA expression was calculated using the FPKM statistics.

### miRNA Mining and Prediction

Data from the venom glands of the sea snake were sampled, and clean reads were obtained by library construction and sequencing in quality control. The filtered sequences were then compared with the miRBase (Griffiths-Jones et al. 2006, 2007) database for known miRNA statistics. Unannotated sequences were used predict new miRNAs. miRNA expression levels were estimated by localizing them to the mature sequences of the species and counting the newly predicted miRNA sequences. Expression counts were performed based on the identified known and newly predicted miRNAs. miRNA expression was calculated using transcripts per million (TPM) (Sun et al. 2014) to calculate the metric.

### lncRNA Mining and Prediction

The rRNA was removed using the whole transcriptome library method as well as SortMeRNA (Kopylova et al. 2012) and trimmomatic (Bolger et al. 2014) software, and the sequencing data were tested using FastQC (de Sena Brandine and Smith 2019) software to ensure the reliable quality of the obtained reads. The quality-controlled clean reads were compared with the reference genome of the corresponding sea snake using HISAT2 (Kim et al. 2019), the proportion of each type of comparison was counted, and the comparison results were evaluated using the RSeQC (Wang et al. 2012) suite. After obtaining the aligned sam files, the reads aligned to the genes were assembled using StringTie (Pertea et al. 2015) software, and the transcripts from each sample were fused and spliced into a complete transcript. The fused transcripts were compared with the known reference genome annotation files of each sea snake by Cuffcompare software, and transcripts with Class code = “u,” “I,” “x,” and “o” in the comparison results were screened. The coding potential of the screened transcripts was predicted based on the following criteria: The lncRNAs do not have the ability to encode proteins, are >200 bp in length, and have a number of exons ≥ 2. The final intersection was considered the result. Four prediction methods, CPC (Kang et al. 2017), CNCI (Sun et al. 2013), Pfam (Sonnhammer et al. 1998), and PLEK (Li et al. 2014), were included.

### Acquisition of Proteomics Data and Bioinformatic Analysis

The extracted venom gland tissues and venom components of the two snakes were partly removed for protein concentration determination and sodium dodecyl sulfate polyacrylamide gel electrophoresis (SDS–PAGE) (supplementary fig. S16, Supplementary Material online) and partly for trypsin digestion. The samples were identified by liquid chromatography-tandem mass spectroscopy (LC-MS/MS) (supplementary table S6, Supplementary Material online) after desalting the digested peptides. In the first step, the traditional data-dependent acquisition (DDA) method was used to establish a protein spectrum library. In the second step, the mass spectral data of each sample were collected using DIA technique, and extraction of quantitative information and subsequent statistical analysis was performed on the basis of the DDA database.

### Identification of Toxins and Related Proteins

According to a previous report by our team, the toxin family database was established by comparing the transcripts in the venom glands of sea snakes with various databases and manually verifying to identify toxins and related proteins in the quantitative proteomics.

### Multiomic Conjoint Analysis

#### Transcriptome–Proteome Correlation Analysis

First, we quantified the transcriptome and proteome of the venom glands. The quantification data of transcriptome were converted into TPM values. Then, the raw abundance values of transcriptome and proteome were normalized using the centered log-ratio (clr) transformation function from the R package compositions. This approach allows for the comparison of expression levels of the same gene between transcriptome and proteome in a relatively standardized dimension, thus enabling an assessment of the correlation between the two omics. The correlation between the two groups was evaluated using three metrics, Pearson correlation coefficient (*R*), Spearman correlation coefficient (*ρ*), and coefficient of determination (*R*^2^). All analyses were conducted in the R software.

#### Expression Consistency Analysis

The mRNAs and proteins in the venom gland were sorted by the quantity of expression from highest to lowest and divided into four ranges, top 25%, 25–50%, 50–75%, and 75–100%. For a toxin-related gene, if there was no change in the range of expression level from the transcriptome to the proteome, it was recognized as a gene with expression consistency. If a change occurred, such as gene x ranking within the top 25% range of expression levels in the transcriptome but within the 25–50% range in the proteome, it was defined as downregulation. The downregulation was linked to the degradation action of mRNA by miRNA. In contrast, the upregulation was considered to be likely due to lncRNA competition for binding miRNA, which weakened the silencing effect of miRNA, thus leading to a relative increase in mRNA translation and protein expression levels. A schematic diagram is shown in supplementary figure S17, Supplementary Material online.

#### Prediction of miRNA-Targeted Genes

The correlation between miRNAs and mRNAs in the three samples was first calculated using the Pearson function based on the expression of miRNAs and mRNAs. The threshold value of the correlation analysis was the absolute value of the correlation coefficient, which was ≥0.80 and the *P* value was ≤0.05. Negatively regulated pairs were screened according to the principle of miRNA–mRNA action. The miranda (Enright et al. 2003) program was used to match the miRNAs with mRNA sequences. The target relationship between the miRNAs and mRNA was predicted synthetically based on the energy stability of miRNA and mRNA binding. The threshold parameters used in the prediction were as follows: S ≥ 140, ΔG ≤ 1 kcal/mol, and demanded strict 5′ seed pairing. A global false discovery rate (FDR) for the correlation coefficient was set to <0.05 in the analysis.

#### Analysis of lncRNA–miRNA Interactions

This method is similar to that used to identify miRNA target genes. The correlation between miRNAs and lncRNAs was calculated to screen for negatively regulated pairs. The binding between these miRNA–lncRNA sequences was predicted according to the miranda program using the default parameters of miranda v3.3a.

#### ceRNA Analysis

Based on the quantitative results of transcriptome, miRNA, and lncRNA profiling, Pearson correlation coefficients were calculated for three sets of pairs: mRNA–lncRNA, mRNA–miRNA, and miRNA–lncRNA. Pairs with a correlation coefficient absolute value of ≥0.80 and a *P* value of ≤0.05 were selected as having a positive correlation for mRNA–lncRNA pairs and a negative correlation for miRNA–lncRNA and mRNA–miRNA pairs. Furthermore, using the principle of complementary base pairing and miranda software, potentially interacting mRNA–miRNA and lncRNA–miRNA pairs were screened. Next, miRNAs were used as a bridge to connect mRNA and lncRNAs using the MuTAME method to predict miRNAs targeted by competing mRNA–lncRNA pairs. The ceRNAScore, calculated by hypergeometric distribution, was used as an indicator of prediction accuracy. Finally, by combining the positively correlated mRNA–lncRNA pairs obtained in the first step with the predicted mRNA–lncRNA pairs exhibiting competitive relationships, we generated multiple sets of mRNA–lncRNA pairs that may compete for binding to the same miRNA, thereby forming a ceRNA regulatory network. The *P* values were adjusted using BH method to control the FDR (<0.05) in the analysis. The schematic diagram is shown in supplementary figure S18, Supplementary Material online.

## Toxin–Receptor Interaction Analysis

The 3FTx toxins expressed in the transcriptome, proteome, and venom proteome of the venom glands of the two sea snake were subjected to 3D structural simulations using AlphaFold2-Colab (Mirdita et al. 2022). Sequences of the nAchRα-7b subunit (Uniport ID: Q7T2U0) of *Takifugu rubripes*, the prey of *H. curtus*, and the European eel (*Anguilla anguilla*), the prey of *H. cyanocinctus*, were then obtained from the National Center for Biotechnology Information (NCBI) database: *nAchRα-2* subunit. (NCBI-Access number: XP_035277668.1). The sequences of the nAchRα-7b subunit were also Blast compared in the nr database to obtain the full-length nAchR sequences of the two sea snake prey. Then, the TMHMM-2.0 (Möller et al. 2001) tool was used to predict the extramembrane sequence fragments, and SignalP-5.0 (Almagro Armenteros et al. 2019) was used to filter out the signal peptide sequences after the extramembrane sequences were extracted. The final sequence of the clean nAchR-α subunit was subjected to structural simulation (AlphaFold2-Colab). The three proposed prey species of *H. cyanocinctus* were *A. anguilla*, *Periophthalmus magnuspinnatus*, and *Oxudercinae*. The nine prey receptors proposed for *H. curtus* are *T. rubripes*, *Scophthalmus maximus-505*, *Scophthalmus maximus-487*, *Alosa sapidissima*, *Tachysurus fulvidraco*, *Silurus meridionalis*, *Siluroidei*, *Clarias magur*, and *Perca fluviatilis*. Finally, the 3FTx toxins of *H. cyanocinctus* and *H. curtus* were docked to their prey receptors using HDOCK SERVER (Yan et al. 2020). A total of four groups of docking scores were obtained: Toxin_Hc versus Prey_Hc, Toxin_Hc versus Prey_Hh, Toxin_Hh versus Prey_Hc, and Toxin_Hh versus Prey_Hh. We utilized the docking score as a criterion for evaluating the magnitude of toxin–receptor-binding affinity. Differences were compared by grouping the sources of the toxins, for example, statistics of whether there is a significant difference between the prey receptors of *H. cyanocinctus* and the prey receptors of *H. curtus* for the toxins of *H. cyanocinctus*. First, the homogeneity of variance between two data sets was checked by *F*-test. If the homogeneity of variance was satisfied, a *t*-test was conducted to determine whether there was a significant difference between the two data sets. If the homogeneity of variance was not met, Welch's test should be used to determine whether there is a significant difference in the activity of toxins from the two different species of sea snakes on their prey receptors.

## Supplementary Material

msad125_Supplementary_Data

## Data Availability

Supplementary material are available at Molecular Biology and Evolution online. The Supplementary Datasets have been deposited in Figshare https://doi.org/10.6084/m9.figshare.22672984). All other data are available from the corresponding author upon reasonable request.

## Supplementary Material

Supplementary data are available at *Molecular Biology and Evolution* online. The Supplementary Datasets have been deposited in Figshare (https://doi.org/10.6084/m9.figshare.22672984).
