# Supplementary material for: Comparative Venom Multiomics Reveal the Molecular Mechanisms Driving Adaptation to Diverse Predator–Prey Ecosystems in Closely Related Sea Snakes
Source: Mol Biol Evol. 2023 Jun 5;40(6):msad125. doi: 10.1093/molbev/msad125 (PMC10265070; doi:10.1093/molbev/msad125)
Supplement: msad125_Supplementary_Data [file msad125_supplementary_data.pdf]

Supplementary Material for

Comparative venom multi-omics reveal the molecular mechanisms driving adaptation to diverse predator-prey ecosystems in closely related sea snakes

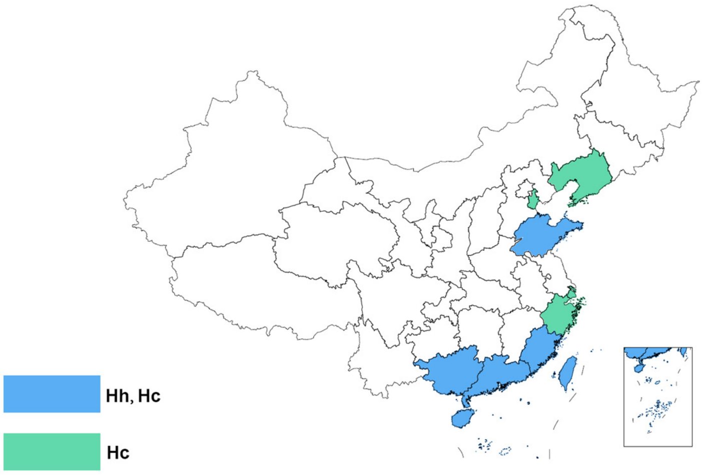

Supplementary Fig. S1. Geographical distribution of the two sea snakes in China. Hc, *H. cyanocinctus* (http://www.sp2000.org.cn/species/show_species_details/6d55e532fb4c4496b4d1f49dcc36acd1); Hh, *H. curtus* (http://www.sp2000.org.cn/species/show_species_details/5058b9a8fb894bf8bbe3aabfefbbd8df).

Supplementary Table S1. Comparison of the morphology and prey between *H. cyanocinctus* and *H. curtus*.

	H. cyanocinctus	***H. curtus***
**Geographical distribution (China)**	Guangxi, Shandong, Fujian, Zhejiang, Hainan, Shanghai, Guangdong, Liaoning, Tianjin, Taiwan, Hong Kong	Guangdong, Guangxi, Hong Kong, Taiwan, Shandong, Fujian, Hainan
**Craniofacial morphology**	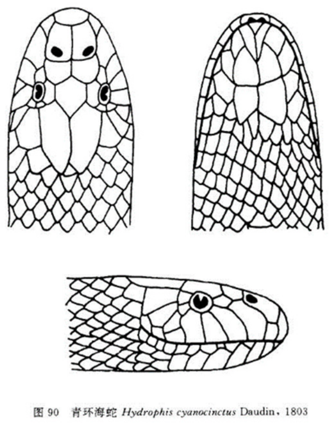	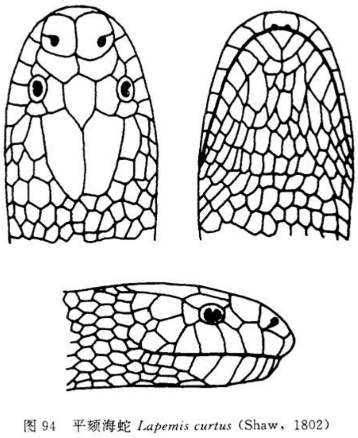
**Gape**	Medium	Relatively larger
**Relative girth (girth at 0.75 SVL : girth at neck)**	1.65 ± 0.116	1.364 ± 0.104
**Prey**	**Anguilliformes (eel)**<br>Ophichthinae (snake eel): *Ophichthus apicalis*, *Cirrhimuraena* sp., *Brachysomophis crocodilinus*;	**Gobiiformes**<br>Eleotridae (sleeper): *Eleotris* sp.; Gobiidae (goby): *Trypauchen* sp., *Taenioides* sp.

	Congridae (conger eel)<br>**Gobiiformes**<br>Gobiidae (goby): *Taenioides* sp. (eel-like goby)	**Anguilliformes (eel)**<br>Moringuidae; Chlopsidae<br>**Perciformes**<br>Sciaenidae; Apogonidae; Siganidae; Callionymidae; Leiognathidae; Gerreidae; Mullidae; Terapontidae; Haemulidae; Trichiuridae; Carangidae; Sparidae; Labridae; Lutjanidae; Nemipteridae; Platycephalidae; Polynemidae; Priacanthidae<br>**Clupeiformes**<br>Clupeidae; Pristigasteridae; Engraulidae<br>**Pleuronectiformes**<br>Cynoglossidae; Soleidae<br>**Siluriformes**<br>Ariidae; Plotosidae<br>**Mugiliformes**<br>Mugilidae<br>**Aulopiformes**<br>Synodontidae<br>**Tetraodontiformes**<br>Tetraodontidae<br>**Syngnathiformes**<br>Fistulariidae<br>**Scorpaeniformes**<br>Scorpaenidae<br>**Decapodiformes**<br>Teuthida; Sepiidae
**References**	(Zhao et al. 1998) (Glodek and Voris 1982) (Voris and Voris 1983) (IUCN 2018) (Sanders et al. 2013) (Sherratt et al. 2018) (Fry et al. 2001) (Lobo et al. 2005)	

Supplementary Table S2. Full names and abbreviations of toxin families.

Abbreviation	Full name
CRISP	Cysteine-rich secretory protein
PLI	Beta-phospholipase A2 inhibitor
DPP-IV	Dipeptidyl peptidase IV
Veficolin	Ficolin lectin family, Veficolin subfamily
PDE	Nucleotide pyrophosphatase/phosphodiesterase
5'-NT	5'-nucleotidase
QPCT	Glutaminyl-peptide cyclotransferase
AChE/CES	Type-B carboxylesterase/lipase

VF	Venom complement C3 homolog
CST	Cystatin
SVSP	Snake venom serine proteases
3FTx	Snake three-finger toxin
vKUN	Venom Kunitz-type
Waprin	Snake waprin
MCO	Multicopper oxidase
GH56	Glycosyl hydrolase 56
PLB	Phospholipase B-like
vLEC	True venom lectin
CATH	Cathelicidin
TCTP	Translationally controlled tumor protein
SVMP	Snake venom metalloproteinases
PLA2	Phospholipase A2
CAL	Calmodulin
NP	Natriuretic peptides
LAAO	L-amino acid oxidase
PDGF	Platelet-derived growth factor

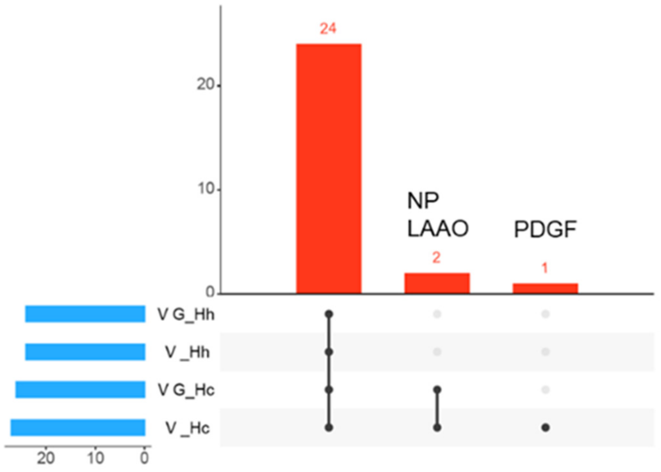

Supplementary Fig. S2. Upset plots of toxins and related proteins in the venom glands and venoms of the two sea snakes.

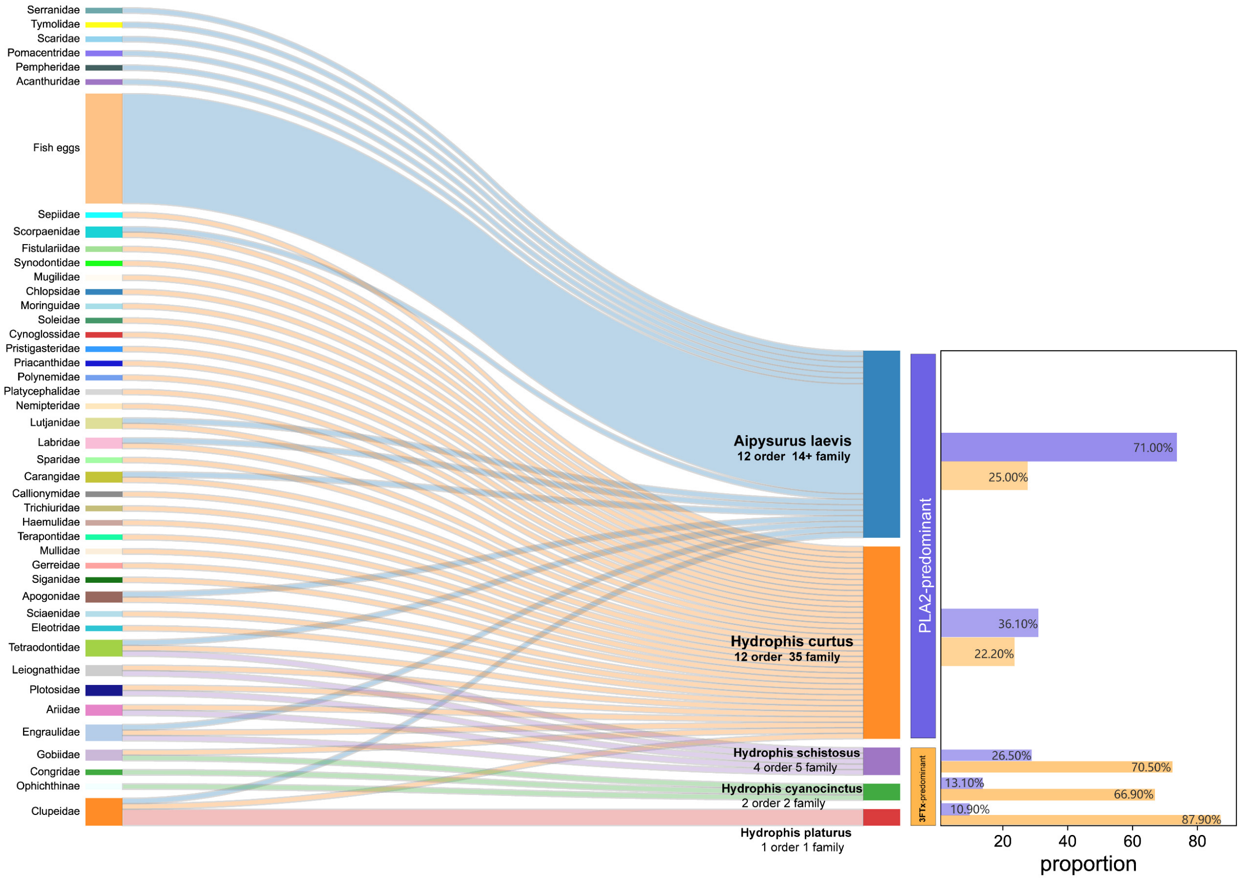

Supplementary Fig. S3. Comparison of the prey diversity and venom proportions of 3FTx/PLA2 in five sea snake species.

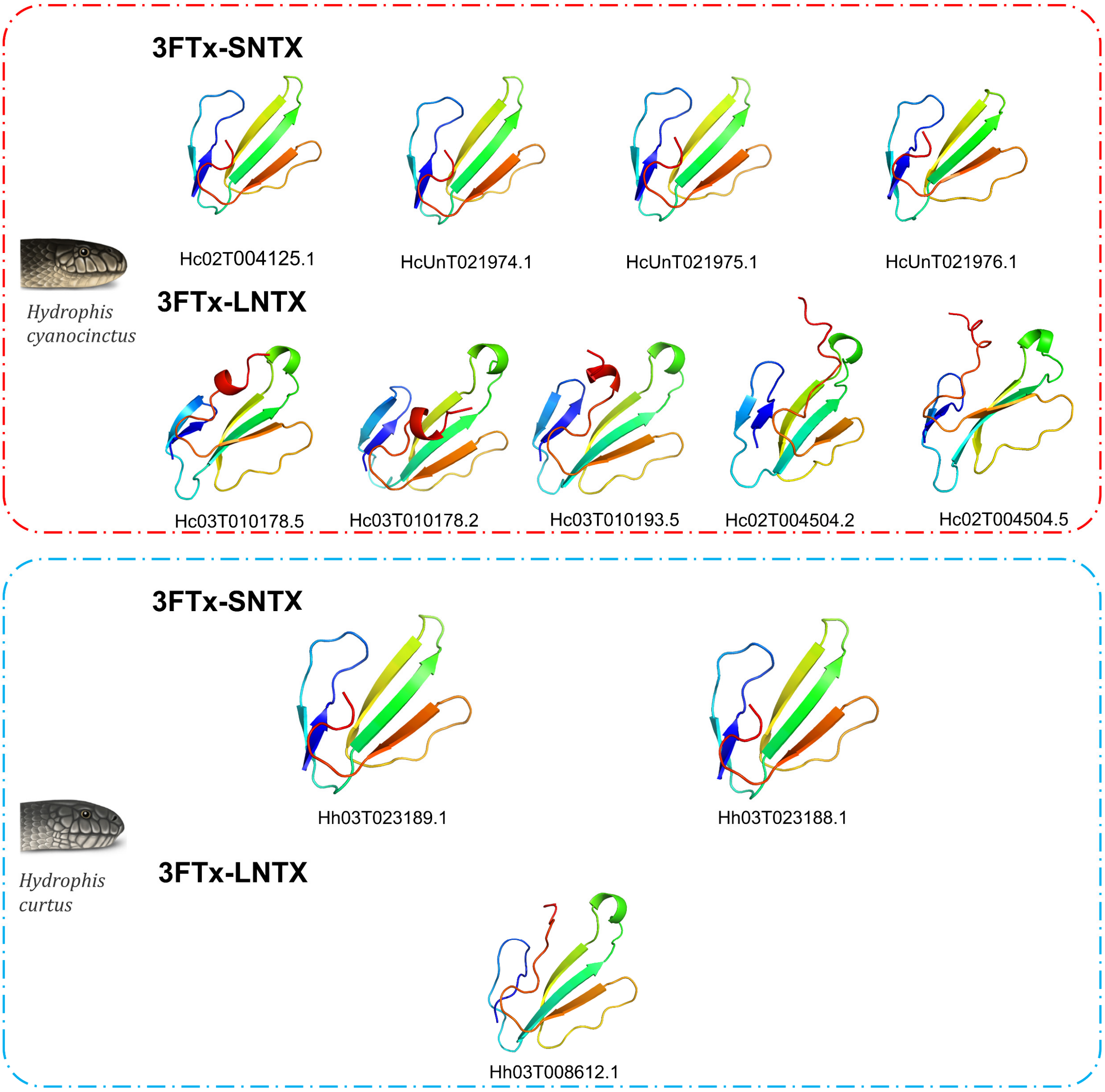

Supplementary Fig. S4. 3FTx structure simulation. 3FTx-LNTX: short-chain three-finger toxins; 3FTx-SNTX: long-chain three-finger toxins; The character below each protein structure picture represents the corresponding protein ID number; *Hydrophis cyanocinctus* detected nine expressed 3FTx, four short-chain neurotoxins and five long-chain neurotoxins. *Hydrophis curtus* detected only three 3FTx, two short-chain neurotoxins and one long-chain neurotoxin.

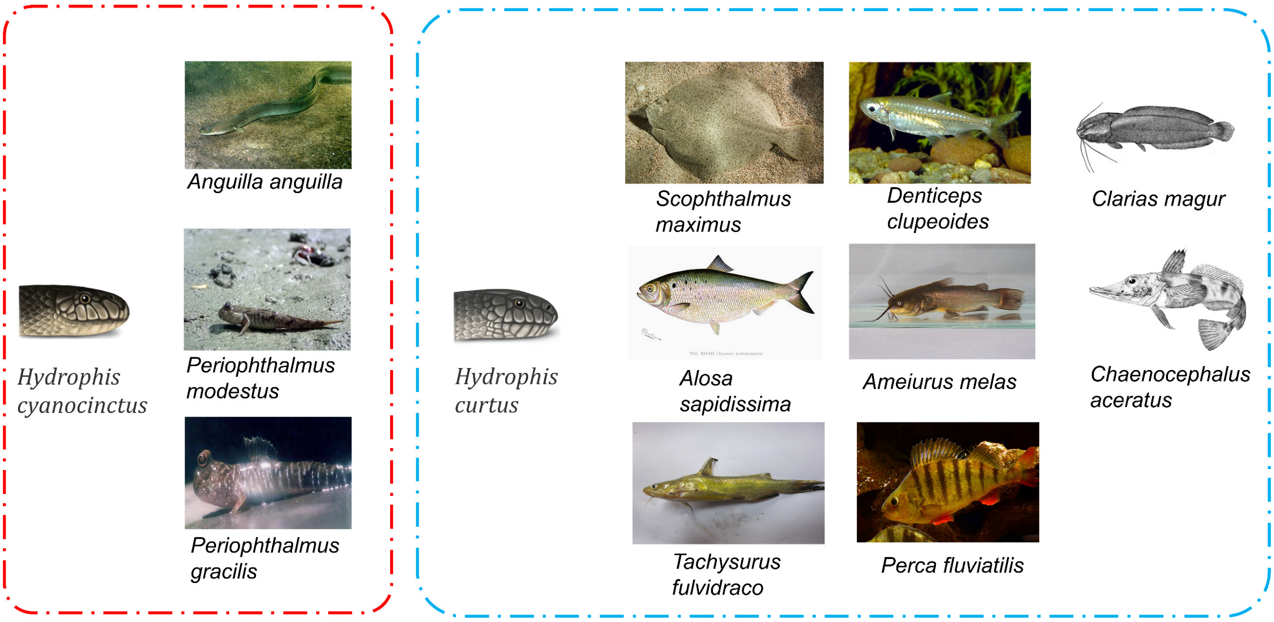

Supplementary Fig. S5. Prey species selected for nAchR docked to 3FTx of the two sea snakes. Sequences of nAchR of these prey will be used for protein structure simulation and 3FTx for docking. Three prey items of *Hydrophis cyanocinctus* were collected, involving Anguilliformes and Gobiiformes. Nine prey items of *Hydrophis curtus* were collected, involving Pleuronectiformes, Clupeiformes, Siluriformes, Perciformes, Clupeiformes.

Supplementary Table S3. *Hydrophis cyanocinctus* 3FTx docking score statistics.

Toxin	*Anguilla Anguilla*	*Periophthalmus magnuspinnatus*	*Periophthalmus gracilis*	*Tachysurus fulvidraco*	*Scophthalmus maximus-505*	*Scophthalmus maximus-487*	*Perca fluviatilis*	*Denticeps clupeoides*	*Clarias magur*	*Chaenocephalus aceratus*	*Ameiurus melas*	*Alosa sapidissima*
Hc02T0041251	-233.27	-251.52	-284.88	-253.53	-252.04	-229.49	-237.73	-234.84	-266.6	-225.64	-223.93	-252.02
Hc02T0045042	-286.59	-261.37	-275.97	-258.91	-261.6	-247.52	-267.73	-249.61	-262	-242.18	-276.43	-256.12
Hc02T0045045	-286.59	-261.37	-275.97	-258.91	-261.6	-247.52	-267.73	-249.61	-262	-242.18	-276.43	-256.12
Hc03T0101782	-333.49	-282.14	-303.98	-290.19	-284.38	-254.51	-280.77	-251.04	-246.8	-281.82	-292	-247.54
Hc03T0101785	-333.49	-282.14	-303.98	-290.19	-284.38	-254.51	-280.77	-251.04	-246.8	-281.82	-223.93	-247.54
Hc03T0101935	-240.37	-251.76	-298.34	-247.5	-258.61	-243.42	-237.5	-259.48	-241.9	-261.65	-284.85	-253.79
HcUnT0219741	-231.82	-232.88	-242	-237.37	-243.59	-237.2	-225.73	-233.51	-227.6	-239.18	-250.1	-241.98
HcUnT0219751	-219.21	-211.36	-238.38	-238.07	-217.5	-220.73	-222.02	-215.95	-223.2	-206.79	-222.09	-221.04
HcUnT0219761	-219.21	-211.36	-238.38	-238.07	-217.5	-220.73	-222.02	-215.95	-223.2	-206.79	-222.09	-221.04

Supplementary Table S4. *Hydrophis curtus* 3FTx docking score statistics.

Toxin	*Anguilla Anguilla*	*Periophthalmus modestus*	*Periophthalmus gracilis*	*Tachysurus fulvidraco*	*Scophthalmus maximus-505*	*Scophthalmus maximus-487*	*Perca fluviatilis*	*Denticeps clupeoides*	*Clarias magur*	*Chaenocephalus aceratus*	*Ameiurus melas*	*Alosa sapidissima*
Hh03T0086121	-222.67	-228.83	-224.04	-305.46	-302.16	-302.13	-301.74	-281.75	-272.05	-274.31	-296.64	-273.10
Hh03T0231881	-193.36	-196.00	-215.81	-253.53	-223.93	-229.49	-237.73	-234.84	-266.61	-225.64	-223.93	-252.02
Hh03T0231891	-193.18	-196.20	-215.40	-224.46	-241.15	-233.71	-217.4	-231.42	-223.97	-214.26	-241.15	-232.39

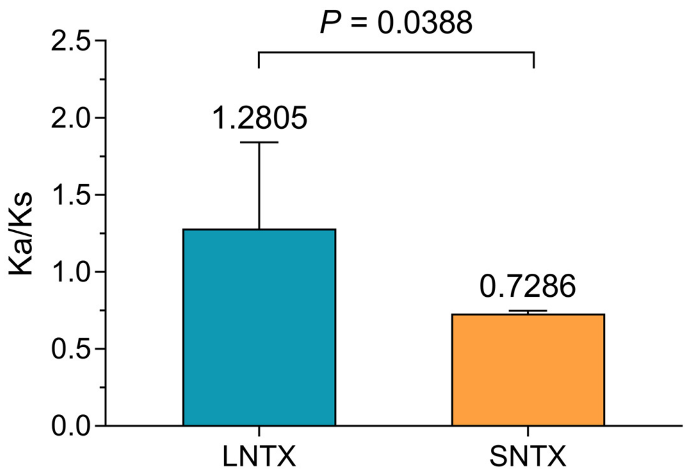

Supplementary Fig. S6. Comparison of Ka/Ks ratios of 3FTx genes in *H. cyanocinctus*. Ka (nonsynonymous substitution rate) and Ks (synonymous substitution rate) values were calculated by KaKs_Caculator2.0 using the GNG model. LNTX, long-chain neurotoxin. SNTX, short-chain neurotoxin. Bars represent mean ± SEM. The significant difference between LNTX and SNTX subfamilies was checked by Mann-Whitney test.

Supplementary Table S5. Clean reads statistics of miRNA sequencing of the two sea snakes.

Sample	raw_reads	reads_trimmed_length	reads_trimmed_Q20	reads_trimmed_N	clean_reads	clean_reads_uniq
Hh_1	26675334	22919252	22909998	22898029	22898029	1643121
Hh_2	20344839	17627330	17619719	17608018	17608018	3571487
Hh_3	35529551	30282733	30263140	30236134	30236134	3151092
Hc_1	33661179	29755106	29734653	29706353	29706353	2935187
Hc_2	28476610	25184273	25174759	25162451	25162451	1924352
Hc_3	27008939	25189772	25172512	25148569	25148569	1860127

Supplementary Table S6. Alignment of Rfam databases for miRNA sequencing of the two sea snakes.

Rfam_type	Number_of_total	%_of_total(%)	Number_of_unique	%_of_unique(%)	Species
rRNA	7692	0.03	1571	0.1	Hh
tRNA	1406	0.01	453	0.03	Hh
snRNA	7144	0.03	2242	0.14	Hh
Cis-reg	7635	0.03	1852	0.11	Hh
other_Rfam_RNA	18386	0.08	3704	0.23	Hh
rRNA	8611	0.03	2212	0.08	Hc
tRNA	2234	0.01	637	0.02	Hc
snRNA	11313	0.04	3410	0.12	Hc
Cis-reg	10533	0.04	2539	0.09	Hc
other_Rfam_RNA	32466	0.11	5243	0.18	Hc

a

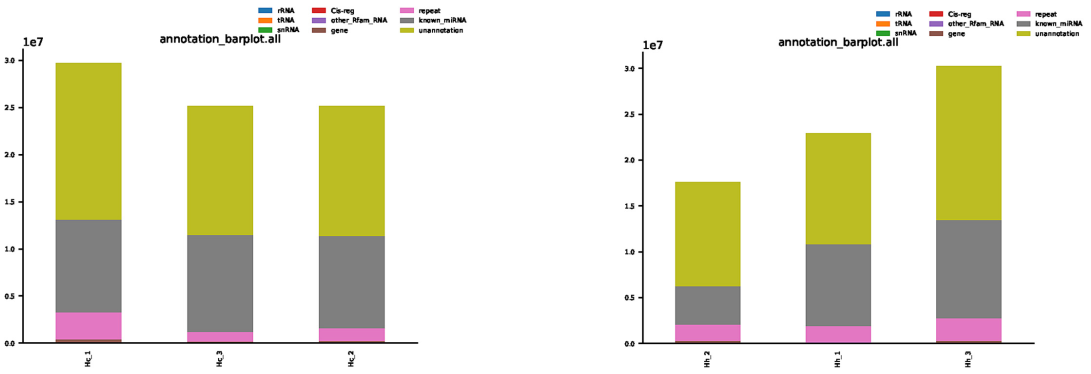

b

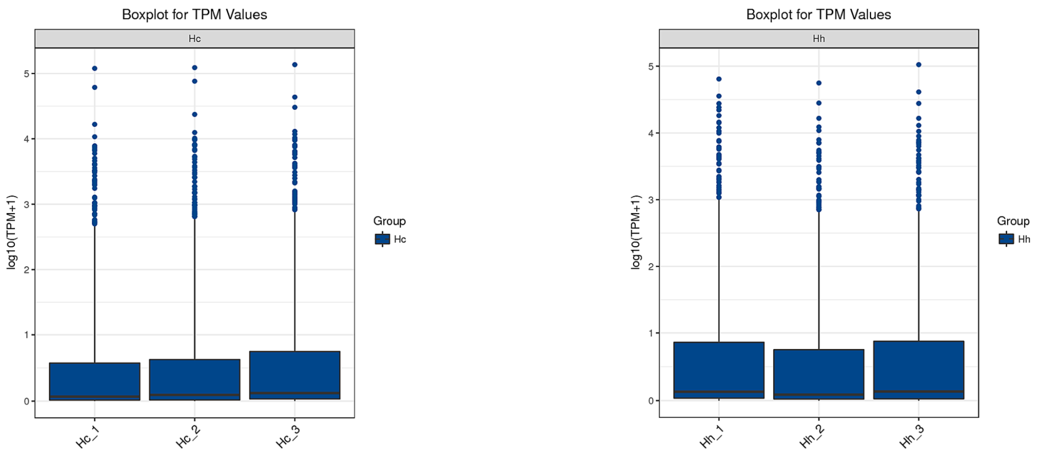

Supplementary Fig. S7. Bioinformatic analysis of miRNA sequencing of the two sea snakes. a. Alignment of Rfam databases for miRNA sequencing of the two sea snakes. The horizontal axis is the sample and the vertical axis is the number of various SmallRNA reads. **b**. Box plots of miRNA expressions of two sea snake. Each species has three sample replicates with consistency between samples.

a

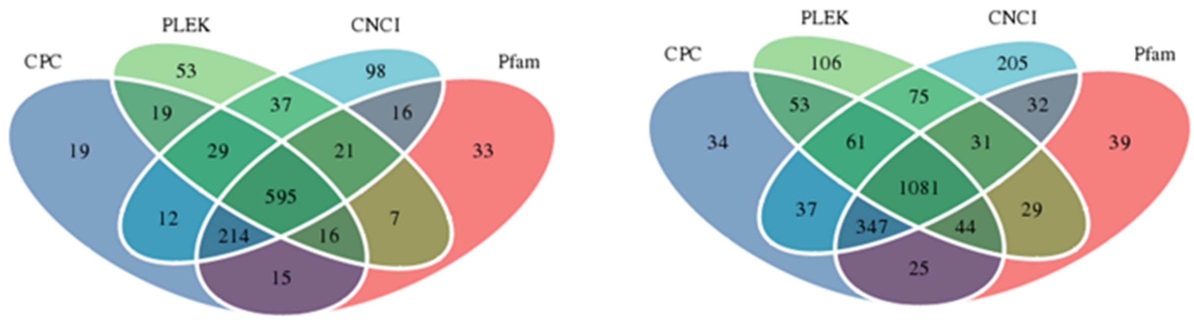

b

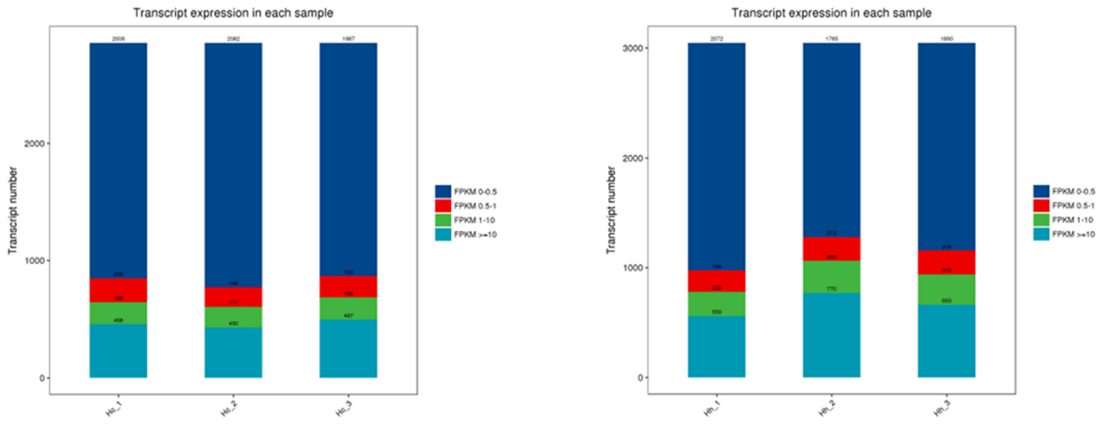

Supplementary Fig. S8. Prediction and expression of lncRNAs in the venom glands of the two sea snakes. **a.** Venn diagram for predicting the amount of lncRNAs using different tools. The left panel shows *Hydrophis cyanocinctus* and the right panel shows *Hydrophis curtus*. **b**. Stacked histogram of the number of transcripts for different regions of the sample lncRNA expression values (FPKM).

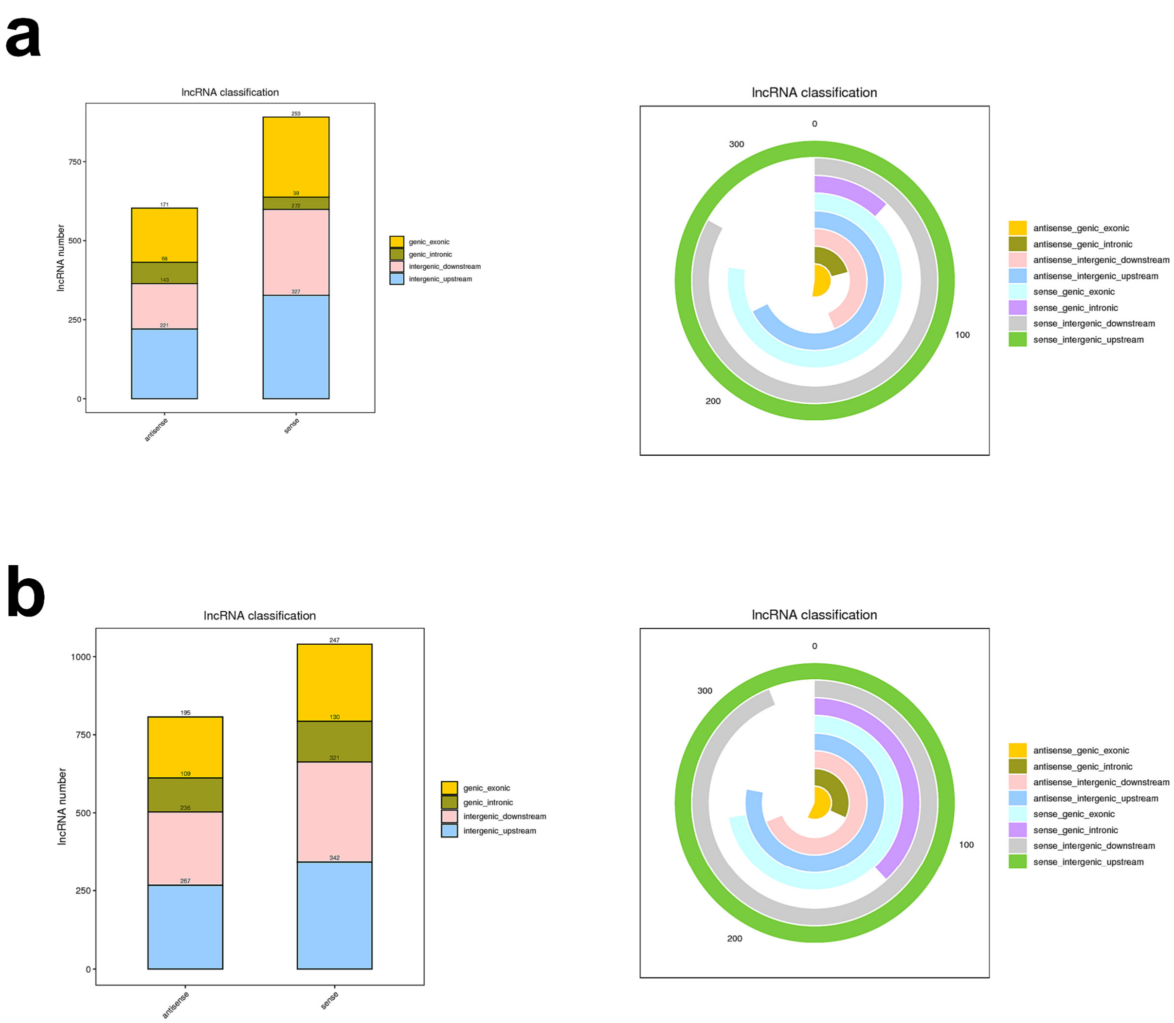

Supplementary Fig. S9. Statistics of lncRNA types predicted in the two sea snake venom glands. The classification criteria were direction, species, and localization. *Hydrophis cyanocinctus* on the upper panel (**a**), *Hydrophis curtus* on the lower panel (**b**).

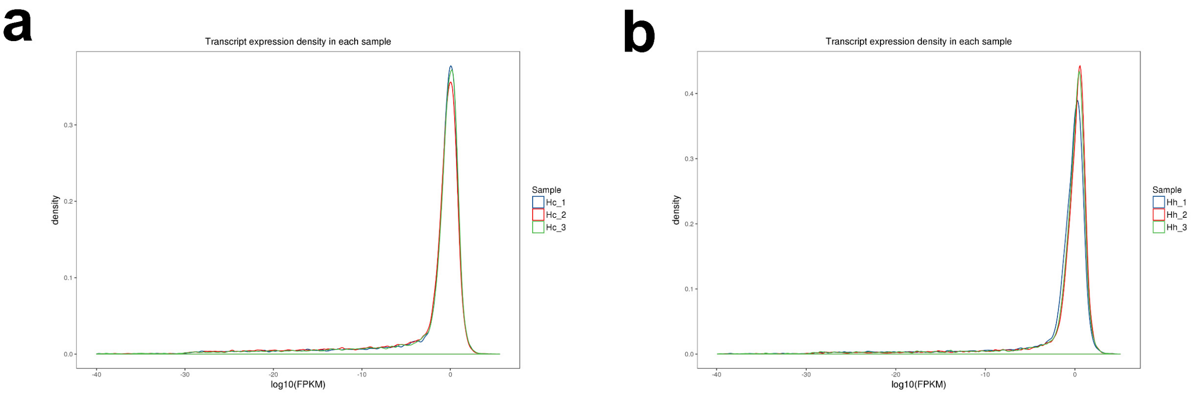

Supplementary Fig. S10. Density distribution of mRNA FPKM values for each sample of the two sea snake venom glands. The curves in different colors represent different samples, the horizontal coordinates of the points on the curves indicate the logarithmic values of FPKM of the corresponding samples, and the vertical coordinates of the points indicate the probability density. The left panel shows *Hydrophis cyanocinctus* and the right panel shows *Hydrophis curtus*.

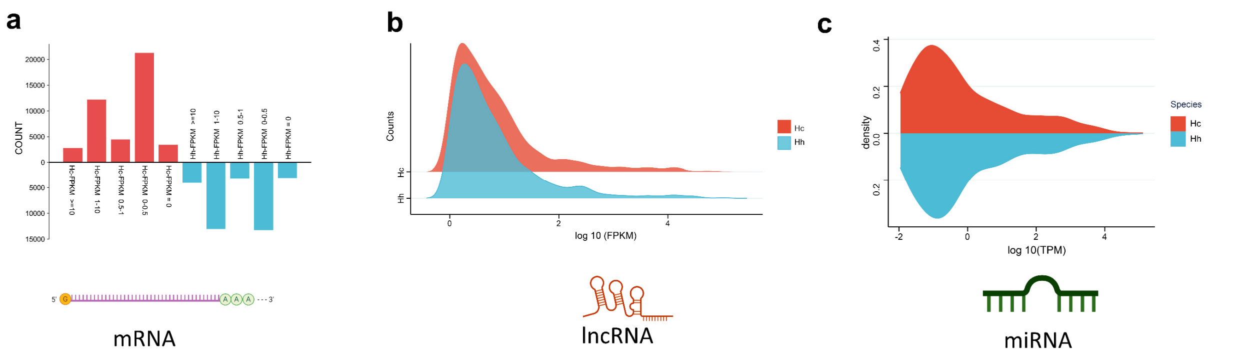

Supplementary Fig. S11. Quantitative data of venom gland multi-omics of *H. cyanocinctus* and *H. curtus*. a, Expression distribution of mRNAs in the venom glands based on FPKM values. **b**, Expression distribution of lncRNAs in the venom glands based on FPKM values processed by $\log_{10}$-transformation. **c**, Expression distribution of miRNAs in the venom glands based on TPM (transcripts per million) values processed by $\log_{10}$-transformation. Three samples of each species were used in the multi-omics analysis. Hc, *H. cyanocinctus*; Hh, *H. curtus*.

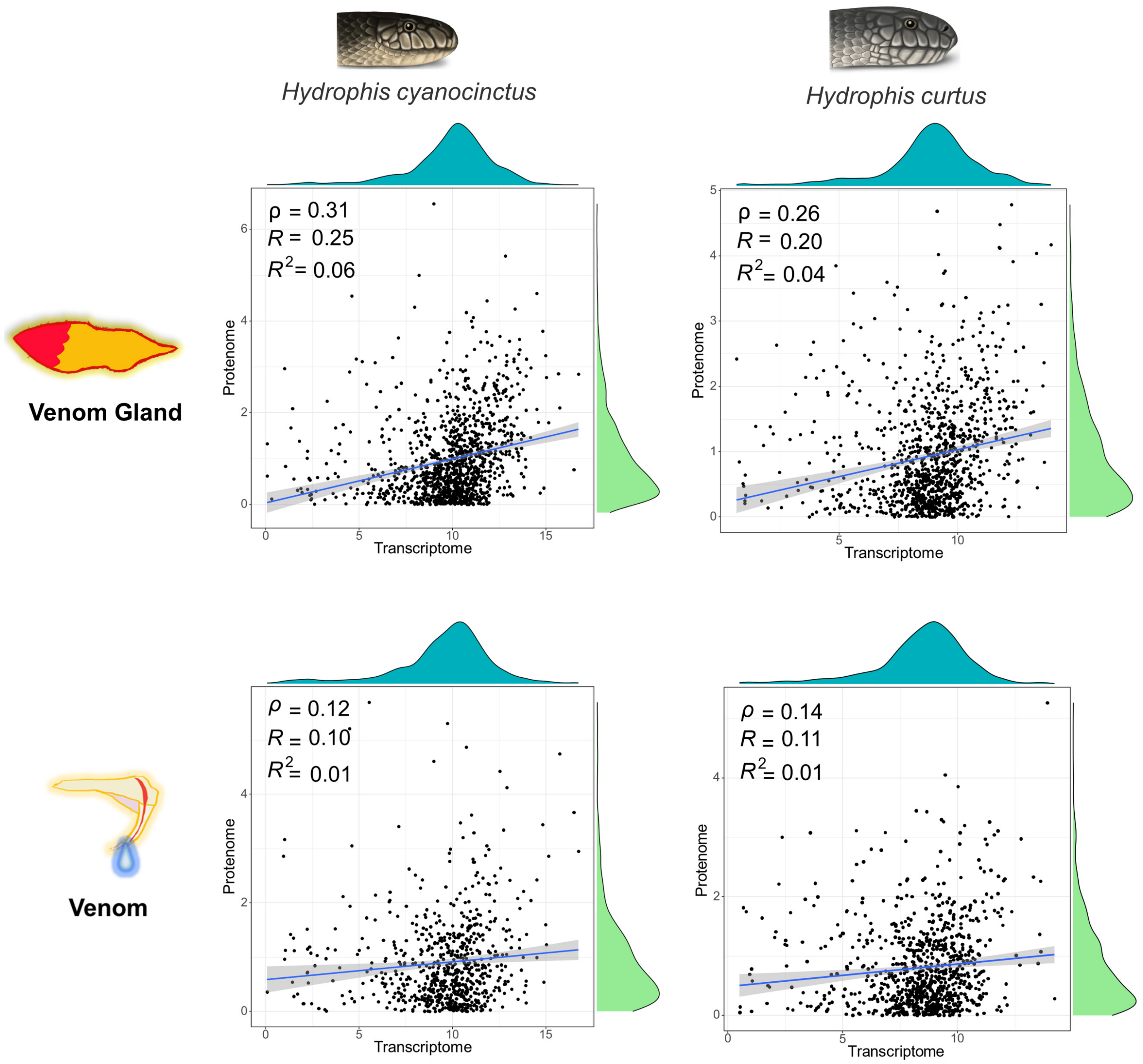

Supplementary Fig. S12. Correlation analysis between venom gland transcriptome, venom gland proteome, and venom proteome expression levels in *H. cyanocinctus* and *H. curtus*.

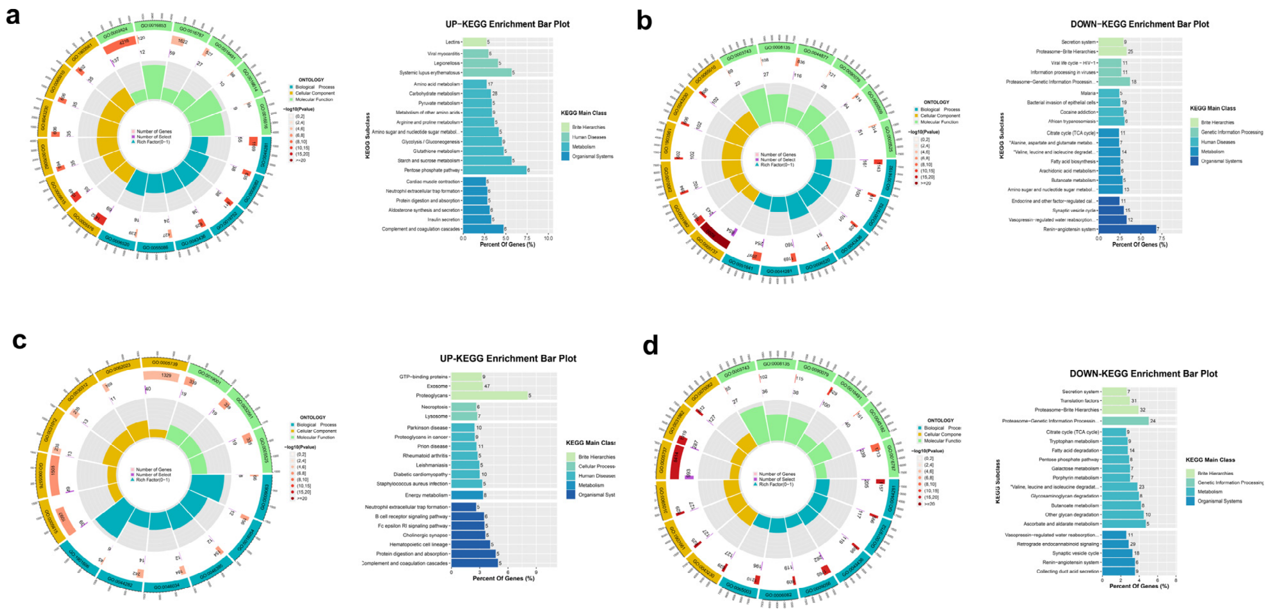

Supplementary Fig. S13. Functional enrichment of up- and down-regulated genes in the venom glands of *H. cyanocinctus* and *H. curtus*. a, Top18 GO annotation map and top20 KEGG annotation map of the up-regulated genes of *H. cyanocinctus*. **b**, Top30 18 annotation map and top20 KEGG annotation map of the down-regulated genes of *H. cyanocinctus*. **c**, Top12 GO annotation map and top20 KEGG annotation map of the up-regulated genes of *H. curtus*. **d**, Top18 GO annotation map and top20 KEGG annotation map of the down-regulated genes of *H. curtus*.

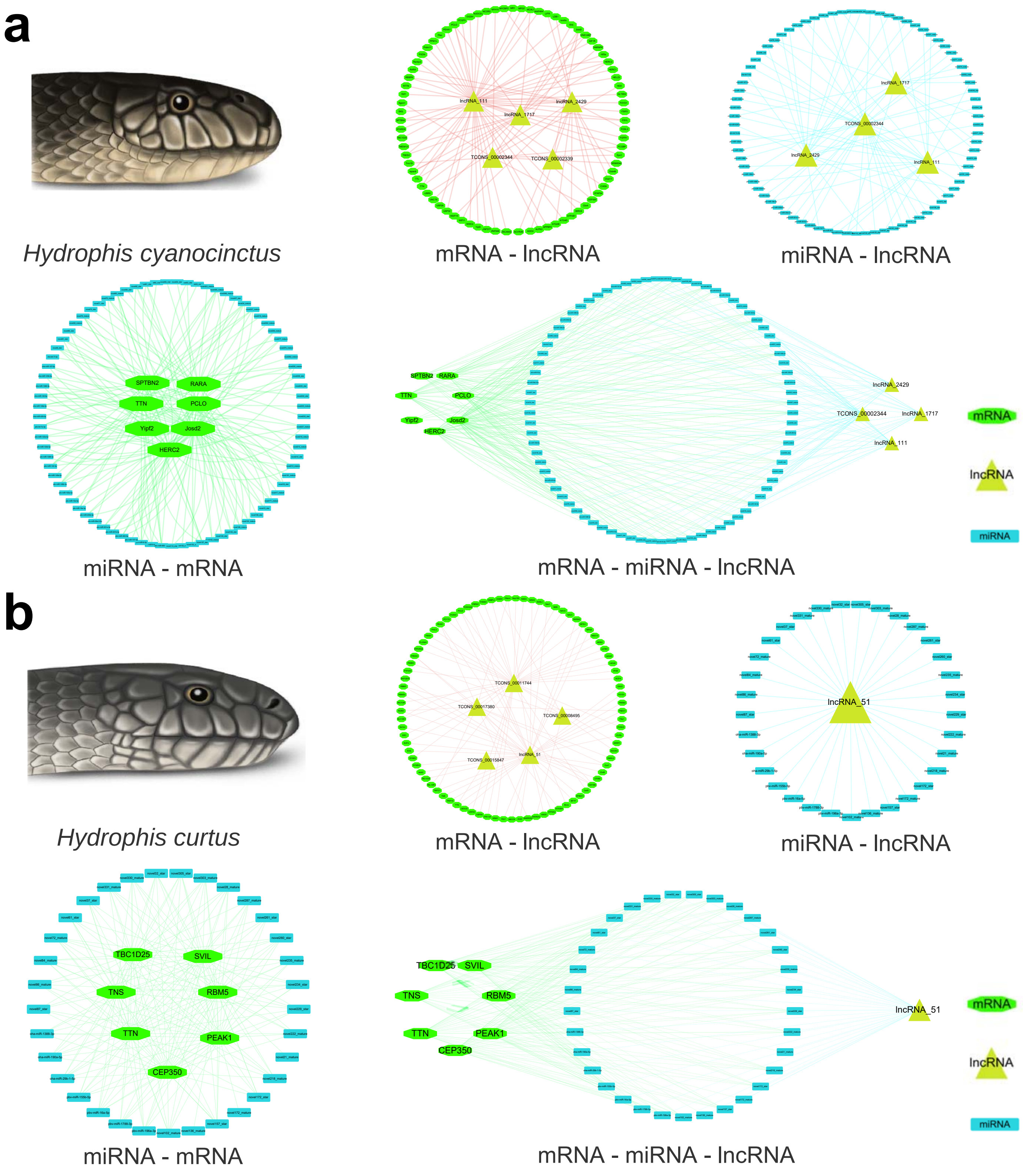
a
Hydrophis cyanocinctus
mRNA - lncRNA
miRNA - lncRNA
miRNA - mRNA
mRNA - miRNA - lncRNA
lncRNA_111
lncRNA_1717
lncRNA_2429
TCONS_00002344
TCONS_00002339
SPTBN2
RARA
TTN
PCLO
Yipf2
Josd2
HERC2
mRNA
lncRNA
miRNA
b
Hydrophis curtus
mRNA - lncRNA
miRNA - lncRNA
miRNA - mRNA
mRNA - miRNA - lncRNA
TCONS_00011744
TCONS_00017380
TCONS_00008495
TCONS_00015847
lncRNA_51
TBC1D25
SVIL
TNS
RBM5
TTN
PEAK1
CEP350
mRNA
lncRNA
miRNA

Supplementary Fig. S14. Regulatory networks of the mRNA-miRNA-lncRNA interactions in the venom glands of *H. cyanocinctus* (a) and *H. curtus* (b).

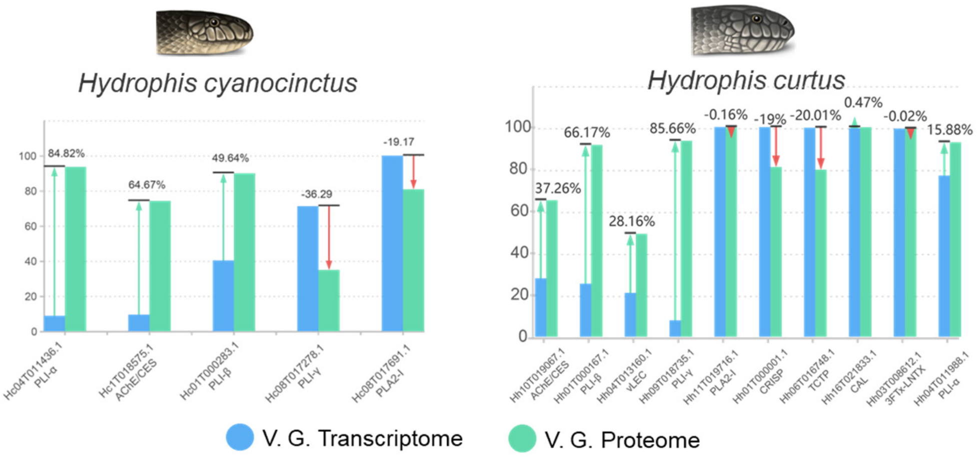

Supplementary Fig. S15. Bar chart of changes in expression levels of major toxin-related genes in the venom glands from the transcriptome to the proteome.

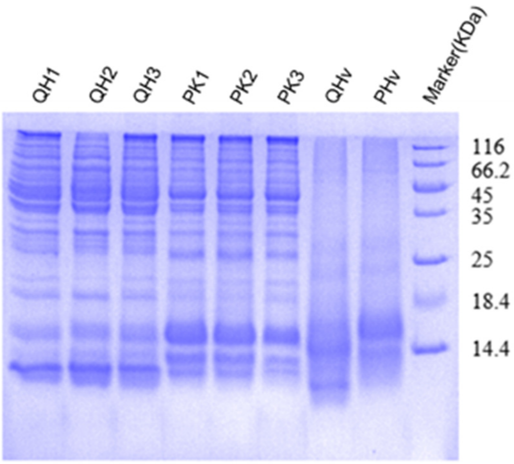

Supplementary Fig. S16. SDS-PAGE electrophoresis of the two sea snake venom gland and venom samples. QH and PK stands for the venom gland of *Hydrophis cyanocinctus* and *Hydrophis curtus*, respectively. QHv and PKv stands for the venom of *Hydrophis cyanocinctus* and *Hydrophis curtus*, respectively.

Supplementary Table S6. LC-MS/MS high resolution mass spectrometry detection.

1. chromatographic conditions

Type	Time （min）	Gradient
DDA	0	5% B
DDA	45	27% B
DDA	50	46% B
DDA	55	100% B
DDA	60	100% B

DIA	0	5% B
DIA	45	27% B
DIA	50	46% B
DIA	55	100% B
DIA	60	100% B
DIA	0	5% B

2.Mass Spectrometry Scanning Parameters

Type	Items	Para.
DDA	Capillary	1.4KV
DDA	Dry Temperature	180℃
DDA	Dry Gas	3.0 L/min
DDA	Mass Range	100-1700 m/z
DDA	Ion Mobility	0.85-1.3
DDA	Collision Energy	20-59 eV
DIA	Capillary	1.4KV
DIA	Dry Temperature	180℃
DIA	Dry Gas	3.0 L/min
DIA	Mass Range	100-1700 m/z
DIA	Ion Mobility	0.7-1.3
DIA	Collision Energy	20-59 eV

3. Database building parameters for data parsing

Type	Items	Para.
DDA	Missed cleavage	2
DDA	Fixed modification	Carbamidomethyl（C）
DDA	Variable modification	Oxidation（M）
DDA	Enzyme	Trypsin/P
DDA	Protein FDR Cut Off	0.01
DDA	Peptide FDR Cut Off	0.01
DDA	PSM FDR Cut Off	0.01
DDA	Database	Hh.rename.proteins.updated.fasta
DIA	Precursor Qvalue cutoff	0.01
DIA	Protein Qvalue cutoff	0.01
DIA	Normalization Strategy	Local Normalization
DIA	Quantity MS-Level	MS2
DIA	Precursor Qvalue cutoff	0.01

4. Absorbance and concentration of the sample to be measured

Samples	Absorbance1	Absorbance2	Absorbance3	Mean absorbance	Measurement concentration（ug/uL）	Real concentration（ug/uL）
QH1	0.38	0.396	0.412	0.396	0.392	47.06
QH2	0.37	0.374	0.37	0.371	0.368	44.11
QH3	0.351	0.359	0.351	0.354	0.35	41.99
PK1	0.392	0.402	0.432	0.409	0.405	48.57

PK2	0.433	0.432	0.431	0.432	0.428	51.37
PK3	0.294	0.272	0.265	0.277	0.273	32.81
QHv	0.289	0.261	0.236	0.262	0.258	2.58
PKv	0.31	0.297	0.32	0.309	0.305	30.54

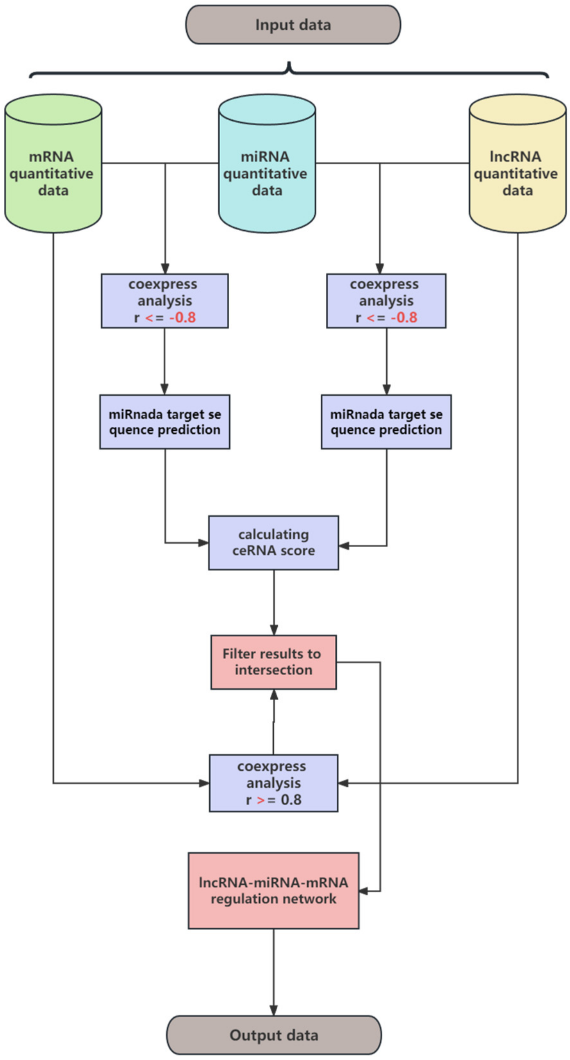

Supplementary Fig. S17. The schematic diagram for ceRNA network analysis.

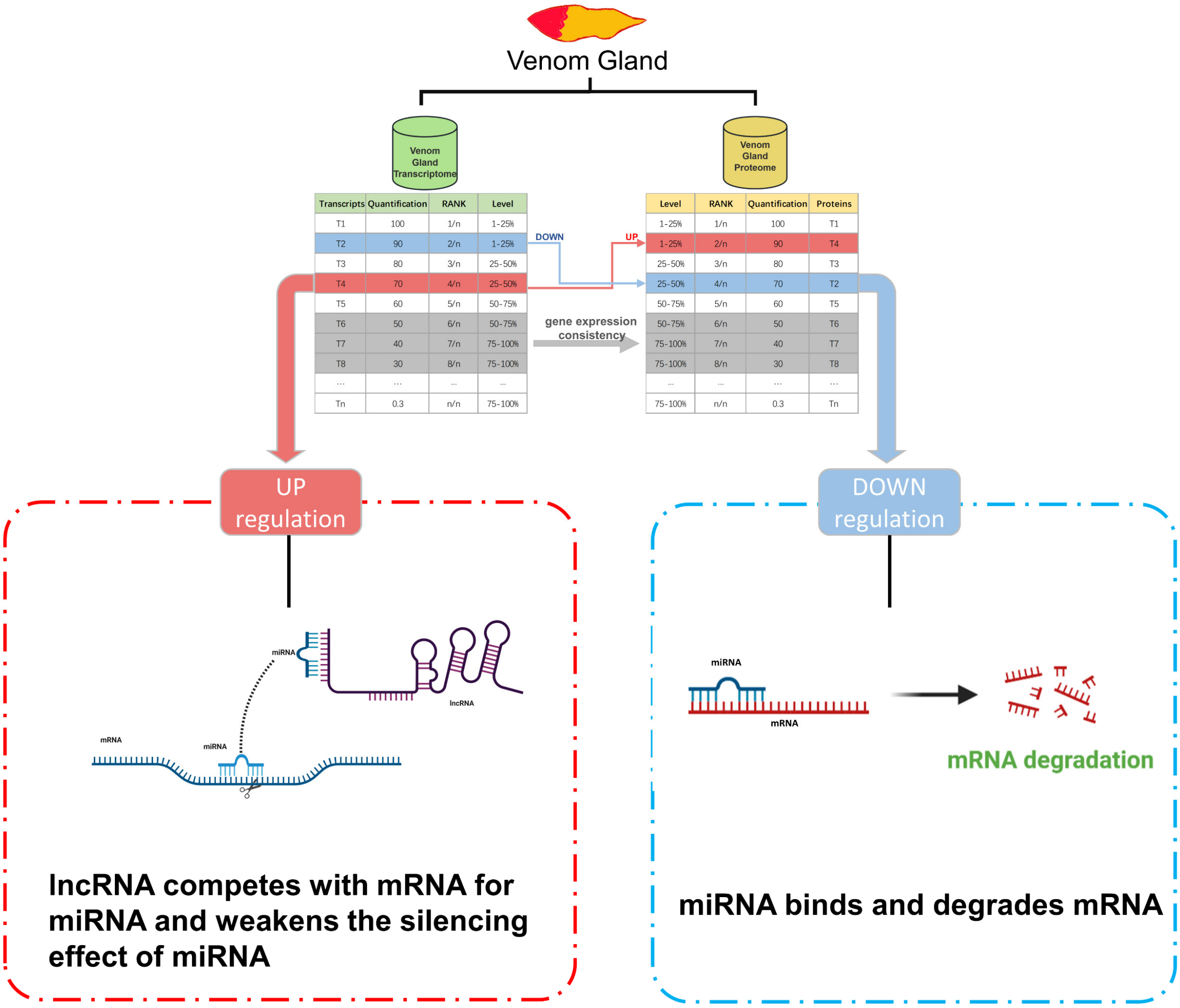

Supplementary Fig. S18. Schematic diagram of the analysis of gene expression consistency and regulation in the venom gland using multi-omics data.
